# Sensitivity to targeted therapy differs between HER2-amplified breast cancer cells harboring kinase and helical domain mutations in *PIK3CA*

**DOI:** 10.1186/s13058-021-01457-0

**Published:** 2021-08-03

**Authors:** Joseph P. Garay, Rebecca Smith, Kaylyn Devlin, Daniel P. Hollern, Tiera Liby, Moqing Liu, Shanta Boddapati, Spencer S. Watson, Amanda Esch, Ting Zheng, Wallace Thompson, Darcie Babcock, Sunjong Kwon, Koei Chin, Laura Heiser, Joe W. Gray, James E. Korkola

**Affiliations:** 1 Department of Biomedical Engineering, Oregon Health & Science University, Portland, OR USA; 2 Department of Genetics, University of North Carolina, Chapel Hill, Chapel Hill, NC USA

## Abstract

**Background:**

*HER2*-amplified breast cancer is a clinically defined subtype of breast cancer for which there are multiple viable targeted therapies. Resistance to these targeted therapies is a common problem, but the mechanisms by which resistance occurs remain incompletely defined. One mechanism that has been proposed is through mutation of genes in the PI3-kinase pathway. Intracellular signaling from the *HER2* pathway can occur through PI3-kinase, and mutations of the encoding gene *PIK3CA* are known to be oncogenic. Mutations in *PIK3CA* co-occur with *HER2*-amplification in ~ 20% of cases within the *HER2*-amplified subtype.

**Methods:**

We generated isogenic knockin mutants of each *PIK3CA* hotspot mutation in HER2-amplified breast cancer cells using adeno-associated virus-mediated gene targeting. Isogenic clones were analyzed using a combinatorial drug screen to determine differential responses to HER2-targeted therapy. Western blot analysis and immunofluorescence uncovered unique intracellular signaling dynamics in cells resistant to HER2-targeted therapy. Subsequent combinatorial drug screens were used to explore neuregulin-1-mediated resistance to HER2-targeted therapy. Finally, results from in vitro experiments were extrapolated to publicly available datasets.

**Results:**

Treatment with HER2-targeted therapy reveals that mutations in the kinase domain (H1047R) but not the helical domain (E545K) increase resistance to lapatinib. Mechanistically, sustained AKT signaling drives lapatinib resistance in cells with the kinase domain mutation, as demonstrated by staining for the intracellular product of PI3-kinase, PIP_3_. This resistance can be overcome by co-treatment with an inhibitor to the downstream kinase AKT. Additionally, knockout of the PIP_3_ phosphatase, *PTEN*, phenocopies this result. We also show that neuregulin-1, a ligand for HER-family receptors, confers resistance to cells harboring either hotspot mutation and modulates response to combinatorial therapy. Finally, we show clinical evidence that the hotspot mutations have distinct expression profiles related to therapeutic resistance through analysis of TCGA and METABRIC data cohorts.

**Conclusion:**

Our results demonstrate unique intracellular signaling differences depending on which mutation in *PIK3CA* the cell harbors. Only mutations in the kinase domain fully activate the PI3-kinase signaling pathway and maintain downstream signaling in the presence of HER2 inhibition. Moreover, we show there is potentially clinical importance in understanding both the *PIK3CA* mutational status and levels of neuregulin-1 expression in patients with *HER2*-amplified breast cancer treated with targeted therapy and that these problems warrant further pre-clinical and clinical testing.

**Supplementary Information:**

The online version contains supplementary material available at 10.1186/s13058-021-01457-0.

## Introduction

The HER2 oncogene is amplified in nearly 25% of all primary breast cancers [1] and has been a focus for targeted therapy development for decades. HER2 is a member of a family of four single-pass transmembrane receptor tyrosine kinases (EGFR (HER1), HER2, HER3, and HER4) that form hetero- and homodimers and activate diverse signaling pathways including phosphatidylinositol 3-kinase (PI3-kinase; reviewed in [2]). The growth and survival of HER2-amplified tumors is dependent on HER2 function, so disruption of HER2 signaling is detrimental to these cells [3, 4]. Several drugs have now been approved that specifically target HER2 signaling, including antibodies such as trastuzumab [5], pertuzumab [6], and small molecules that target the tyrosine kinase activity such as lapatinib [7], tucatinib [8], and neratinib [9]. Ado-trastuzumab emtansine has also been approved, but has a distinct mechanism of action by delivering a cytotoxic agent conjugated to trastuzumab to specifically target HER2-positive cells [10].

HER2-mediated PI3-kinase activation occurs through the recruitment of a 110-kDa catalytic subunit (encoded by PIK3CA) via a p85α regulatory subunit (encoded by PIK3R1) to the cell membrane. This complex activates signaling via phosphorylation of the membrane lipid PIP_2_ (phosphatidylinositol (4,5) bisphosphate) at the 3′ position resulting in phosphatidylinositol (3,4,5) triphosphate (PIP_3_). PIP_3_ serves as a docking site for proteins that contain pleckstrin homology (PH) domains, such as the AKT family of proteins [11]. AKT proteins influence a variety of processes inside the cell involving cell growth, regulation of apoptosis, glucose metabolism, and others [reviewed in [12]].

Importantly, responses to HER2-targeted drugs are thought to be affected adversely by the co-occurrence of mutations that influence downstream PI3-kinase signaling [13, 14]. PIK3CA is the most frequently mutated gene in this pathway with mutations occurring in about 20% of HER2-amplified breast cancers [15]. PIK3CA mutations occur preferentially in two “hotspot” regions of the protein. One hotspot region lies in the kinase domain (H1047R) and the other in the helical domain of *PIK3CA* (E542K and E545K) that binds the p85 regulatory subunit of PI3K [16]. Helical and kinase domain mutations account for about 85% of all mutations seen in *PIK3CA* in breast cancer [17]. These mutations are sometimes combined in clinical outcome association studies [18, 19] based in part on laboratory studies showing that overexpression of the two mutation types in biological models produces similar response phenotypes [20, 21]. However, they are functionally distinct with crystallographic studies of p110α showing conformational differences between the two hotspot mutations [16, 22].

We sought to better understand the impact of helical (E545K) and kinase (1047R) domain mutations on the therapeutic response of HER2+ cells to targeted inhibitors. We have previously reported generation of cell lines in which *PIK3CA* mutations are overexpressed by conventional transgene expression and that *PIK3CA* mutant cell lines show increased resistance to lapatinib [21]. These cells required combination therapy using lapatinib plus an AKT inhibitor to restore sensitivity to the same levels as control cells. However, this methodology is limited because transgenic cells retain two endogenous wildtype copies of *PIK3CA* as well as an unknown number of transgenic mutant alleles. We report here the generation of isogenic knockin mutants of each *PIK3CA* hotspot mutation, which has the major advantage of maintaining *PIK3CA* gene expression under control of the endogenous promoter, resulting in physiological levels of expression of the mutants. This is distinct from the majority of studies that have introduced mutations via transfection or transduction, which result in overexpression of the mutants. We report below our findings of the substantial differences of the impact of kinase versus helical domain mutations of PIK3CA on signaling and therapeutic responses to HER2-targeted receptor tyrosine kinase inhibitors in the HER2-amplified cell line, SK-BR-3.

## Methods

### Plasmids and AAV production

Plasmids for adeno-associated virus (AAV) production containing either E545K or H1047R mutation of PIK3CA were a generous gift of Dr. Ben Ho Park. AAV production was done in HEK293T cells and infection of SK-BR-3 cells with AAV-PIK3CA was performed as previously described [23]. pSpCas9(BB)-2A-Puro (PX459) was a gift from Feng Zhang (Addgene plasmid # 48139) and the protocol for generating knockout cells has been described [24]. CRISPR guideRNA sequences to target PTEN have been designed previously [25].

### Cell lines and compounds

All cell lines were purchased from ATCC (Manassas, VA), cultured in the prescribed medium, and incubated at 37 °C in 5% CO_2_. Cell line identity was confirmed by genotyping, and all cultures were tested to ensure absence of mycoplasma infection. SK-BR-3 selection conditions were 0.5 mg/mL for G418 and 0.25μg/mL for puromycin. Lapatinib, neratinib, the AKT inhibitor GSK690693, and the MEK inhibitors GSK1120212 and PD0325901 were purchased from Selleck Chemicals (Houston, TX).

### DNA isolation and sequencing

Genomic DNA was isolated from cells using the Qiagen (Germantown, MD) DNeasy Blood & Tissue Kit. Primers for PCR amplification and sequencing are listed (Supplemental Table 2). Prior to sequencing, all amplified PCR products were gel isolated on Invitrogen 2% agarose (Thermo Fisher Scientific (Waltham, MA)) gels to ensure purity.

### Cell growth assays

Cells were seeded in Corning (Corning, NY) 96-well white-walled clear-bottom plates. Drug was added 24 h after seeding. Within each plate, each drug concentration was randomly arrayed in triplicate using a liquid-handling robot as described previously [26]. Cell Titer-Glo (Promega (Fitchburg, WI)) was used according to the manufacturer’s recommendations to determine number of viable cells per well at time zero and after 72 h. Dose-response curves for each drug were plotted and used for GR50 and GR_max_ calculations [27]. Each dose-response curve was generated on three separate occasions. For NRG1β-lapatinib response studies, cells were plated in 96-well black-walled clear-bottom plates (Corning). NRG1β EGF Domain (R&D Systems (Minneapolis, MN)) was added 24 h after cell plating, followed immediately by treatment with vehicle or lapatinib at four different concentrations (50, 100, 250, or 500 nM). Cells were incubated for 72 h, fixed with paraformaldehyde, stained with DAPI, imaged on an InCell 6000 (GE (Boston, MA)) instrument, segmented, and analyzed for cell counts following treatment.

### Immunoblot analysis

Cells were harvested in lysis buffer (40 mM HEPES, 75 mM NaCl, 2 mM EDTA, 1% NP-40), spun at 4 °C, and the supernatant collected. Lysate concentration was quantified by Pierce BCA Protein Assay (Thermo Fisher Scientific). Samples were separated by electrophoresis on 4–12% Bis-Tris polyacrylamide gels, transferred to nitrocellulose membrane, blocked for 1 h in Li-Cor (Lincoln, NE) Odyssey buffer, and incubated with primary antibody overnight at 4 °C. All antibody information can be found in Supplemental Table 3. Secondary antibodies were purchased from LiCor, IRDye® 680RD donkey anti-mouse (925-68072), and IRDye® 800CW goat anti-rabbit (925-32211). Membranes were incubated for 1 h with secondary antibody and imaged on the Odyssey 9120 Infrared Imaging System (Li-Cor).

### PIP3 staining

An antibody for PIP3 staining (Echelon Biosciences (Salt Lake City, UT)) was used as previously described [28]. SK-BR-3 cells and derivatives were seeded in 12-well plates with each well containing sterilized glass coverslips. Cells were grown for 24 h to allow them to adhere to the coverslips. After 24 h, drug or vehicle was added to each well and cells were incubated for an additional 72 h. Cells were fixed in 4% paraformaldehyde for 15 min, washed in PBS with 0.5% Tween for 5 min, permeabilized in 2% saponin, and then washed in PBS with 0.5% Tween. Coverslips with adhered cells were blocked in 10% goat serum for 1 h then washed three times in PBS with 0.5% Tween. Primary antibody was diluted in 5% goat serum and coverslips were incubated with primary antibody solution overnight at 4 °C. Coverslips were then washed three times in PBS with 0.5% Tween and incubated with Alexa Fluor® 594-conjugated secondary antibody (Thermo Fisher Scientific) and DAPI for nuclear stain for 1 h at room temperature. Stained coverslips were mounted on microscope slides with VECTASHIELD (Vector Laboratories (Burlingame, CA)) and imaged on the Zeiss 880 confocal microscope. Images were quantified using CellProfiler as described in Fig. 5B.

### Analyses of HER2-enriched samples in public datasets

Two publicly available datasets—one from The Cancer Genome Atlas (TCGA) and one from the Molecular Taxonomy of Breast Cancer International Consortium (METABRIC)—were restricted to samples of the HER2 subtype. For TCGA data, differentially expressed genes in HER2+ tumors in TCGA samples between mutant classes were identified using Significance Analysis for Microarrays (SAM) [29] using the open-source software Multiple Experiment Viewer. Boxplot analysis of individual gene expression and protein expression was performed using GraphPad Prism version 5.0.

### *PTEN* RT-PCR

RNA was isolated from cells using the Zymo Research (Irvine, CA) Direct-zol RNA MiniPrep kit. All samples were diluted to 20 ng/μL and used with Applied Biosystems OneStep RT-PCR kit (Thermo Fisher Scientific). Primers for RT-PCR are listed (Supplemental Table 1). TATA-binding protein (TBP) was used as internal control for RT and PCR steps.

### Statistical analyses

We calculated GR50 values for three separate lapatinib dose-response curves for each of the different cell line mutations. We used standard two-sided *t*-tests to test if GR50 values differed significantly between controls and mutants, with significance at *p* < 0.05, and also applied *t*-tests to identify differential expression of PIP_3_ following lapatinib treatment. Significance in TCGA and METABRIC samples was calculated using Mann-Whitney tests to compare expression between the two different tumor populations. We also calculated synergy using the combination index approach of Chou and Talalay, as described previously [21]. Synergy was deemed to be significant when the upper 95% confidence interval of the combination index was less than 1, as previously described [21].

## Results

### Generation of *PIK3CA* E545K and H1047R knockin mutants

**Fig. 1:**
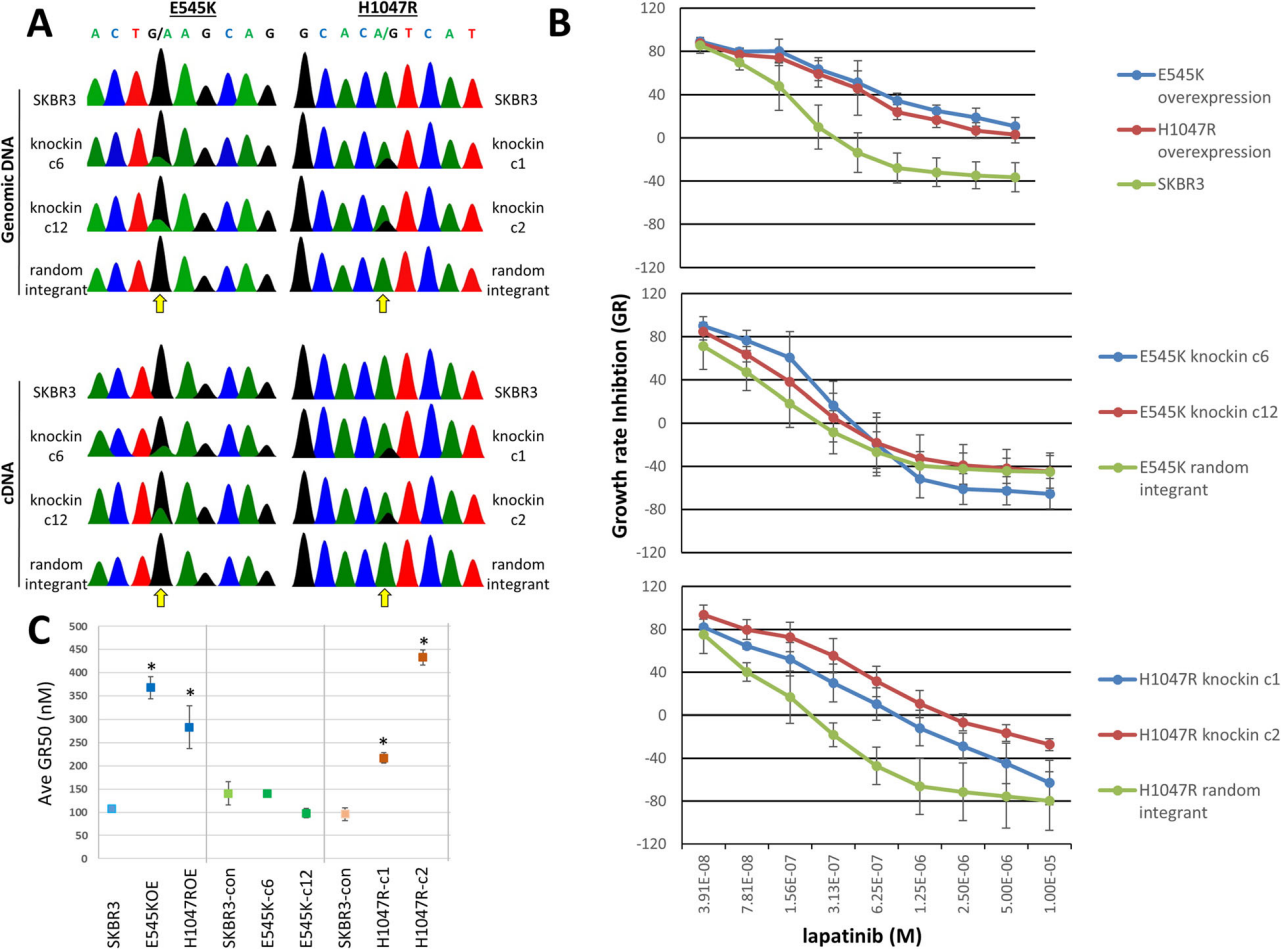
Gene targeting validation in SKBR3 cells and response of knockin derivative cells to lapatinib treatment. **A** Targeted Sanger sequencing of E545K locus (left) and H1047R locus (right) on both genomic DNA (top) and cDNA (bottom) shows correct targeting in two clones for each mutation (arrows). **B** Drug response to lapatinib after 72 h shows that overexpression of *PIK3CA* mutations (top panel) makes cells less sensitive to lapatinib treatment regardless of which mutation is introduced. Site-specific genome editing reveals that while the E545K mutation does not confer reduced sensitivity to lapatinib treatment (middle panel) whereas the H1047R mutation does (bottom panel). Error bars represent standard deviation of triplicate assays. **C** GR50 calculations for SK-BR-3 parental, control, and mutant clones shows that overexpression clones and H1047R mutant knockin clones have significantly higher GR50 values than parental and control cells, but that E545K knockin mutant clones do not. Error bars represent standard deviation of triplicate measurements. * denotes significant difference from control or parental cells by *t*-test (*p* < 0.05)

We developed a realistic model of *PIK3CA* mutation in the HER2+, luminal subtype breast cancer cell line SK-BR-3 by using adeno-associated virus (AAV) vectors to deliver sequences for homology-mediated recombination at the *PIK3CA* locus as previously described [23]. We chose this approach because it puts the mutated *PIK3CA* under endogenous promoter control which can reveal pathological phenotypes that are masked in experiments using overexpression of the same mutant transgene [30]. After infection, antibiotic selection, and single cell cloning, we isolated multiple unique SKBR3 clones and sequenced both genomic DNA and cDNA (Fig. 1A). Clones designated as “random integrant” are cells that were infected with the targeting vector, selected under antibiotic, and single cell diluted but showed only wildtype sequence at the *PIK3CA* locus by analysis of both genomic DNA and cDNA (Fig. 1A) implying that the targeting vector integrated randomly elsewhere in the genome. We used Sanger sequencing to estimate allele frequency in cells (Supplemental Figure 1A). SK-BR-3 cells are nearly triploid throughout the genome and triploid at the genomic locus for *PIK3CA*. Figure 1A shows that targeted knockin of these *PIK3CA* mutations results in alteration of one of three alleles. We also compared the knockin cells to SK-BR-3 cells into which we had previously introduced mutated PIK3CA using retroviral transduction [21]. We found that these retrovirally transduced cells had three copies integrated for the E545K mutant transgene and two copies integrated for the H1047R mutant transgene and overexpressed mutant *PIK3CA* at the protein level (Supplemental Figure 1B and 1C). In contrast, our AAV-targeted knockin lines maintained protein expression levels that were equivalent to wildtype parental cells (Supplemental Figure 1C).

### *PIK3CA* E545K and H1047R knockin mutants respond differently to lapatinib

We tested the knockin clones for their responses to lapatinib since our previous work demonstrated that *PIK3CA* mutations confer resistance to lapatinib monotherapy [21]. Cells harboring the knockin H1047R mutation showed significant resistance to lapatinib (Fig. 1B). We calculated the normalized growth rate inhibition (GR50)—a robust metric for cellular growth response to drug which accounts for cell division rate (Fig. 1C) [27]. The GR50 concentration for lapatinib in SK-BR-3 non-targeted knockin control cells lies within the nanomolar range (96.0 ± 13.6 nM), similar to the parental cells (107.4 ± 6.4 nM), but the two clones with the knockin H1047R mutation had a significantly higher GR50 values (217.2 ± 11.7 nM, *p* < 0.05 compared to control; and 433.0 ± 16.0 nM, *p* < 0.005 compared to control). Interestingly, clones with the knockin E545K mutation remained sensitive to lapatinib and did not show significant differences in response compared to control cells (Fig. 1B, C) (knockin control, GR50 = 140.5 nM ± 25.2 nM; E545K c6 GR50 = 139.7 ± 8.0 nM, *p* = 0.95; E545K c12 GR50 = 98.2 ± 10.1 nM, *p* = 0.09). In contrast, cells engineered to overexpress either the H1047R or E545K mutation of *PIK3CA* were highly resistant to lapatinib. The respective GR50 values for lapatinib in the E545K overexpressing cells and H1047R overexpressing cells were significantly higher than the control SK-BR-3 cells at 368.0 ± 23.9 nM (*p* < 0.005) and 283.0 ± 45.3 nM (*p* < 0.05) respectively (Fig. 1C). We further assessed the growth of mutant clones using continuous live-cell imaging for cells exposed to 500 nM lapatinib over a 90-h period. This dose of lapatinib did not inhibit the growth of cells overexpressing either of the mutant *PIK3CA* transgenes or the H1047R knockin mutants to the same extent as parental and control cells. However, E545K knockin mutants were growth inhibited to similar levels as parental and control cells (Supplemental Figure 2A). We also assessed the inhibitory effects of neratinib on the growth of these engineered cell lines. Neratinib, like lapatinib, is a dual inhibitor of tyrosine kinase activity of EGFR and HER2 but is much more potent and can be used at low nanomolar concentrations. The responses of cells treated with neratinib were similar to those seen with lapatinib (Supplemental Figure 2B). These results suggest that pharmacological inhibition of HER2 signaling can be subverted by kinase domain (H1047R) mutations but by not helical domain (E545K) mutations in HER2-amplified cells.

**Fig. 2:**
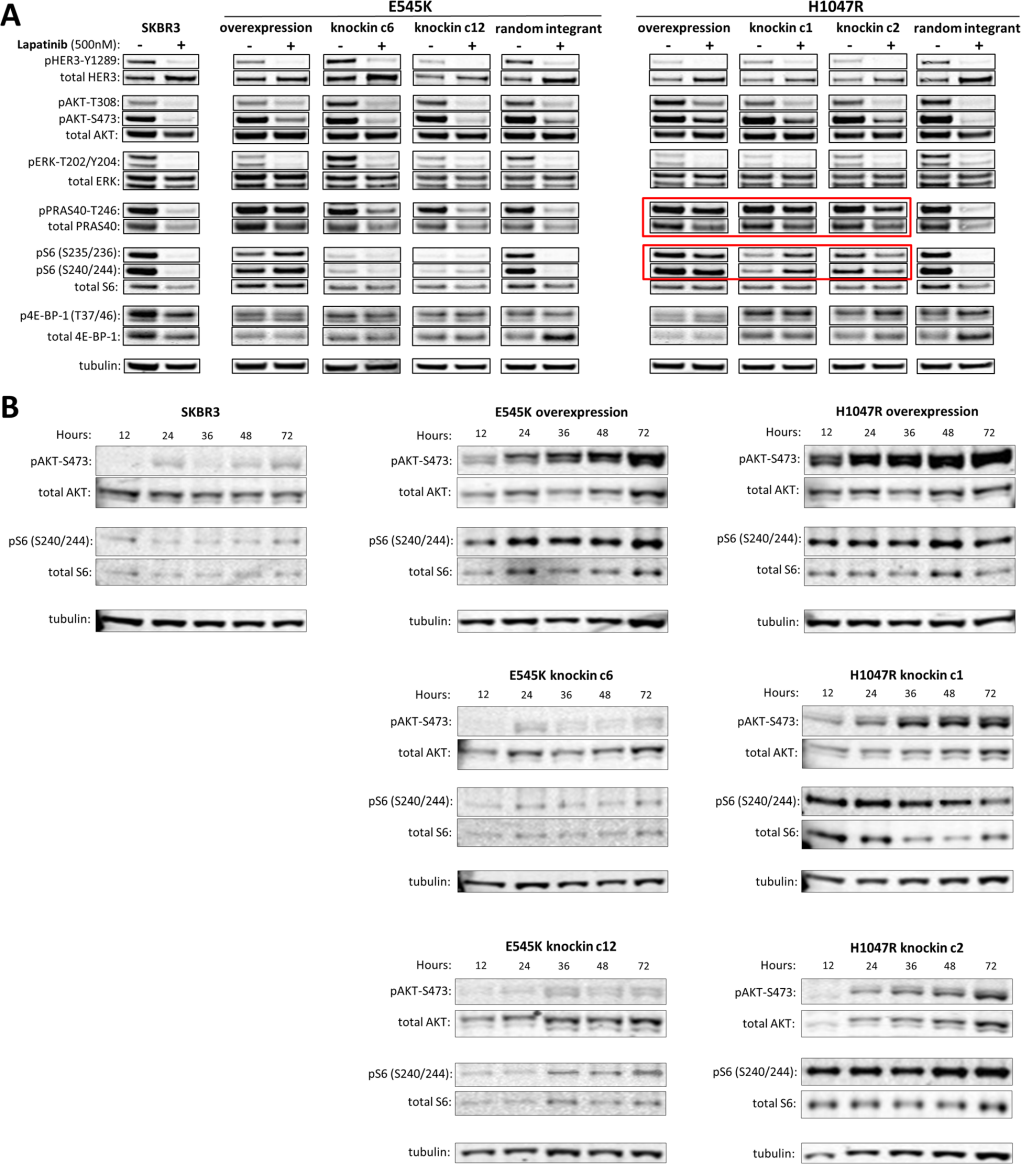
Analysis of signaling pathways showing H1047R knockin cells maintain intracellular signaling in presence of lapatinib. **A** Whole cell lysates collected after 48 h of exposure to 500 nM lapatinib or vehicle were separated by gel electrophoresis and analyzed for the indicated proteins. H1047R knockin cells retain high levels of phospho-AKT and downstream substrates phospho-PRAS40 and phospho-S6 (red boxes) whereas E545K knockin cells do not. **B** Cells were treated with 500 nM lapatinib for a timecourse of 72 h. Whole cell lysates were collected at the indicated times and separated by gel electrophoresis and analyzed for the indicated proteins. H1047R knockin cells show recovery of phospho-AKT levels and persistent phosphorylation of S6 which is not seen in E545K knockin cells

We interrogated cellular signaling pathways via western blot analysis to identify differences in signaling associated with each mutation (Supplemental Table 2). PI3K is primarily activated by HER-family receptor dimerization and transphosphorylation, particularly between HER2 and HER3 which form the most potent signaling dimers. Phosphorylation of HER3, EGFR, and downstream signaling molecules were assessed after 48 h of treatment with lapatinib in all samples. There was an observable difference between the helical (E545K) and kinase (H1047R) domain mutation knockin clones in the phosphorylation of downstream PI3K pathway proteins. Specifically, levels of AKT phosphorylation at serine-473 remained high in H1047R mutants but were reduced in E545K mutants (Fig. 2A, Supplemental Figure 2C). In addition, the level of p-S6 ribosomal protein was higher in the H1047R than in the E545K knockins (Fig. 2A, red boxes), although baseline expression of p-S6 also appeared to be higher in the H1047R mutants. PRAS40 phosphorylation differences were less striking, although it still appeared that there were slightly higher levels of p-PRAS40 in the H1047R mutants than the E545K mutants. However, levels of phospho-4E-BP-1, another downstream target of mTORC1, were not modulated by treatment with lapatinib (Fig. 2A), consistent with our previous observations [21]. Importantly, the clones overexpressing the H1047R and E545K mutants did not show differences in PI3K signaling following treatment with lapatinib. These clones expressed high levels of phospho-AKT, phosphorylation of PRAS40 at serine-246, and phosphorylation of ribosomal protein S6 at Ser-235/236 and Ser-240/244 after treatment with lapatinib for 48 h, similar to what we observed in the H1047R knockin clones (Fig. 2A).

Previous reports have shown that HER2-amplified cells can recover from pharmacological inhibition of HER2 signaling over time even in the presence of drug [31]. Our results after 48 h of treatment are concordant with previously published data showing that concentrations of lapatinib above 200 nM are able to completely block this recovery [31]. However, this observation does not preclude the possibility that knockin H1047R cells or overexpression mutants recover faster than their counterparts. Thus, we examined changes in phosphoprotein levels at multiple times during a 72-h time course treatment with 500 nM lapatinib (Fig. 2B). Cells were replenished with fresh medium and drug every 24 h for the duration of the experiment, ensuring that effects of drug half-life in culture were minimized. Unlike untreated cells which showed strong PI3K signaling activity (see Fig. 2A), we found that PI3K signaling was suppressed within the first 12 h of lapatinib treatment in overexpression clones and in the H1047R knockins. PI3K signaling re-emerged with increased levels of pAKT and pS6 appearing within the next 12 h in kinase domain mutant cells. In contrast, suppression of phosphoprotein signal persisted for much longer in the E545K knockin mutants. In the case of phospho-AKT, this suppression persists for the duration of the timecourse. For phospho-S6, we observed slight recovery of signal in the E454K knockins after 36–48 h, but the level of recovery was diminished compared to recovery in H1047R knockin clones. However, this is complicated by the fact that the baseline levels of phospho-S6 are lower in the E545K clones than the H1047R clones (see Fig. 2A).

### AKT inhibition restores sensitivity to lapatinib in the *PIK3CA* H1047R knockin mutants

**Fig. 3:**
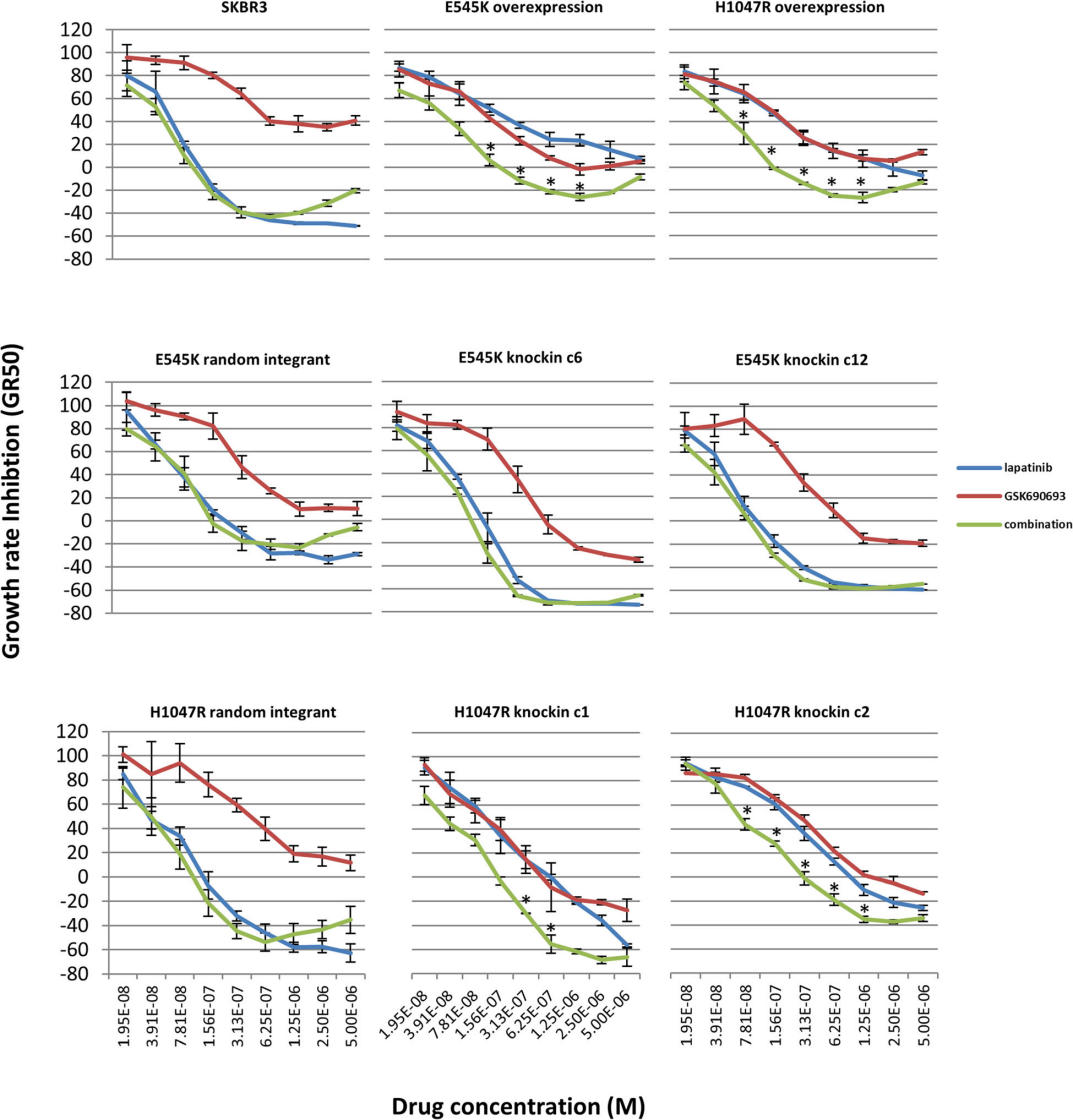
Response of knockin derivative cells to AKT inhibition alone or in combination with lapatinib. Drug response after 72 h shows minimal differential sensitivity to AKT inhibition with GSK690693 (red) among all cell lines. Equimolar combination of both GSK690693 and lapatinib (green) shows synergistic growth inhibition in cells overexpressing *PIK3CA* mutations or with knockin of the H1047R mutation but not the E545K mutation. Points at which significant synergistic interactions as measured by the combination index between lapatinib and GSK690693 are indicated with an *. Lapatinib monotherapy data from Fig. 1B is repeated here in each graph for comparison (blue)

We assessed the efficacy of AKT inhibition in cells after treatment with the pan-AKT inhibitor GSK690693. *PIK3CA* mutant cell lines, whether overexpression or knockin, maintained sensitivity to GSK690693 in the micromolar range by measure of GR50 (Fig. 3 and Supplemental Figure 3A). It has been reported that inhibition of AKT results in a feedback loop that upregulates phospho-AKT-mediated signaling [32]. All cells treated with high-dose GSK690693 (500 nM) showed upregulation of phosphorylated AKT at phospho-Thr308 and phospho-Ser473 sites (Supplemental Figure 3B). This is expected, as ATP-competitive inhibitors of AKT also prevent dephosphorylation of AKT [33]. Phosphorylation of PRAS40 and ribosomal protein S6 also were decreased (Supplemental Figure 3C), demonstrating that GSK690693 as a single agent does inhibit AKT signaling by blocking propagation of its intracellular signal through mTORC1 to ribosomal protein S6.

Because H1047R knockin mutant cells exhibit continual signaling downstream of AKT and treatment with GSK690693 sufficiently blocks signaling downstream of AKT, we sought to test the efficacy of lapatinib and AKT inhibition as a combinatorial therapy. We assayed our panel of cells with varying equimolar amounts of these two drugs using the same cell growth assay as with monotherapy alone. We measured synergistic interactions using combination index measurements, and interactions were deemed significant when the combination index was less than 0.7 and the upper 95% confidence interval was less than 1. We found co-treatment of H1047R mutant cells with both lapatinib and GSK690693 resulted in significantly lower GR50 compared to either monotherapy alone and a restoration of sensitivity to lapatinib (Fig. 3), which is manifested as significant synergy in the H1047R mutant cells. No synergistic growth inhibition with co-treatment of drugs was seen in the wild-type cells or for E545K knockin clones because lapatinib as monotherapy is already highly effective (Figs. 1B and 3 and Supplemental Figure 4). Importantly, lapatinib and GSK690693 were synergistic in both retrovirally overexpressing E545K and H1047R mutants, consistent with our previous findings [21]. We attribute the synergism in the overexpressing E545K mutant to the supraphysiologic level of PI3-K signaling in this clone, where the inhibition of AKT restores sensitivity to lapatinib to the same levels as in wildtype cells.

It has been reported that cells can bypass AKT inhibition by utilizing signaling through the Ras-Raf-MEK-ERK pathway [34, 35], so we further assessed the possibility that cells bypass lapatinib control by utilizing ERK signaling. We addressed this by measuring responses to combined lapatinib and MEK inhibitors. Co-treatment of cells for 72 h with two separate MEK inhibitors—PD0325901 and GSK1120212 (Supplemental Figure 5A)—revealed no synergistic, or even additive, effects in either wildtype or mutant cells. Whole cell lysate analysis of cells treated with either MEK inhibitor alone showed complete loss of phospho-ERK signal after 24 h demonstrating that these compounds have the desired target effects in SK-BR-3 cells (Supplemental Figure 5B). Together, these results imply that these HER2-amplified cells do not rely on the Ras-Raf-MEK-ERK axis for their sustained growth under lapatinib monotherapy, consistent with the reported reliance on PI3K signaling in luminal HER2+ cells [36].

### PTEN knockout phenocopies the drug response of *PIK3CA* H1047R mutants

**Fig. 4:**
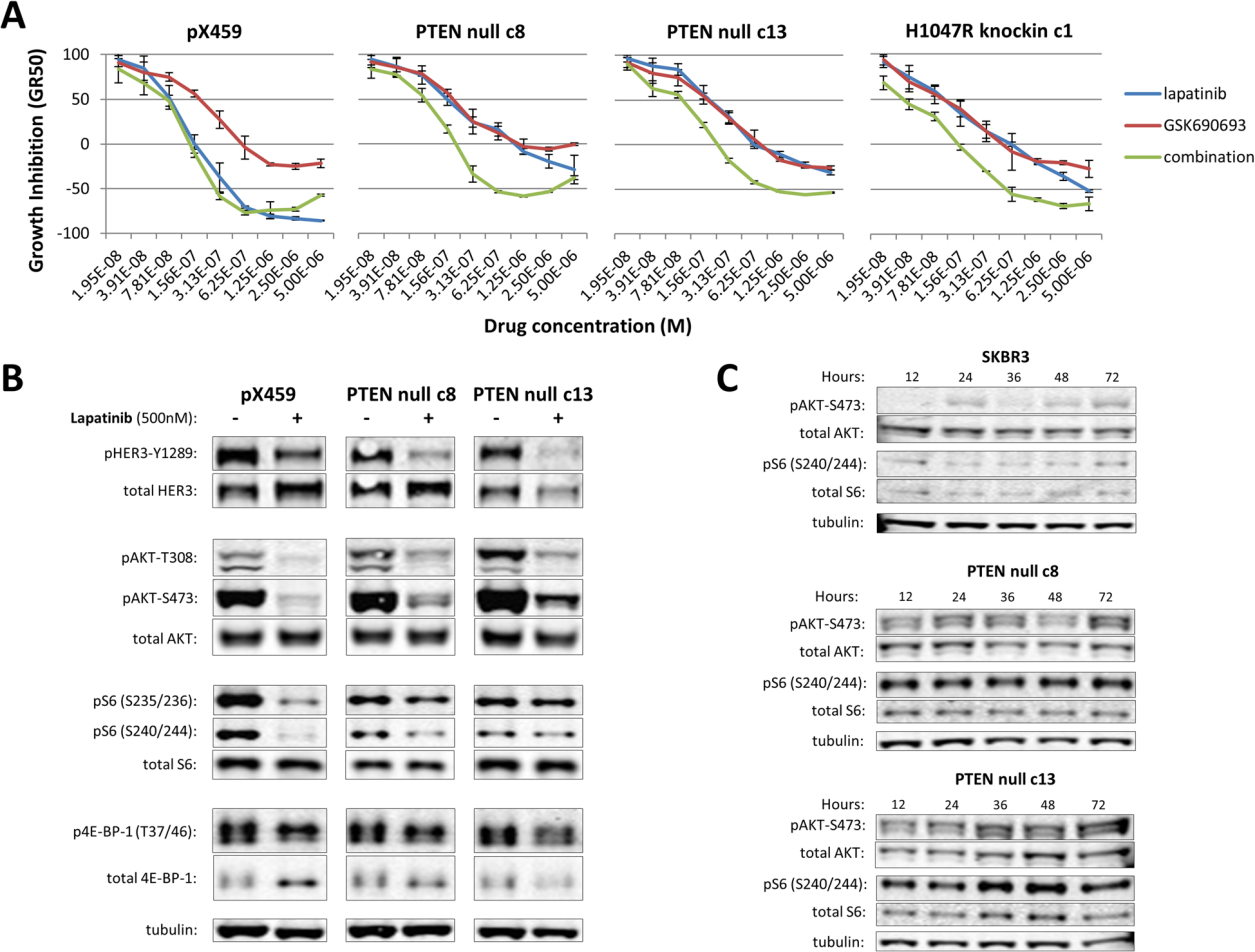
Characterization of PTEN null clones reveals these cells closely phenocopy cells with H1047R mutation. **A** Combinatorial drug screen using lapatinib (blue), AKT inhibitor GSK690693 (red), or equimolar combination of both drugs (green) shows that knockout of *PTEN* makes cells less sensitive to lapatinib treatment, similar to H1047R knockin cells. **B** Whole cell lysates collected after 48 h of exposure to 500 nM lapatinib or vehicle were separated by gel electrophoresis and analyzed for the indicated proteins. **C** Cells were treated with 500 nM lapatinib for a timecourse of 72 h. Whole cell lysates were collected at the indicated times and separated by gel electrophoresis and analyzed for the indicated proteins

We further assessed the role of canonical PI3K-AKT signaling on resistance to lapatinib by exploring the role of PTEN knockout on lapatinib response. PTEN is the phosphatase protein responsible for the negative regulation of PI3K activity; it dephosphorylates the substrates of PI3K, most importantly the dephosphorylation of PI(3,4,5)P_3_ to PI(4,5)P_2_. We used CRISPR-Cas9 and a previously published guide RNA [25] to knockout *PTEN* in SK-BR-3 cells. We isolated two unique clones and validated loss of PTEN by RT-PCR and western blot analysis (Supplemental Figure 6A and 6B). In both clones, sequence analysis of the PTEN locus showed that CRISPR-Cas9 targeting resulted in deletion of base pairs from the coding sequence causing a frameshift and introduction of a premature stop codon (Supplemental Figure 6C). As a control, cells were also transfected with the Cas9 plasmid containing no guide RNA—designated as “pX459.” Additional targeted sequencing of the top three off-target genomic sites for the given guide RNA—as determined by an online CRISPR Design Tool (http://tools.genome-engineering.org) [24]—shows that all potential off-target sites remain wildtype (Supplemental Figure 6D). We tested PTEN null cells for response to lapatinib using the same drug dilution/combinatorial treatment experiment done with the knockin clones. PTEN null cells showed reduced sensitivity to lapatinib alone, as observed for the H1047R knockin clones (Fig. 4A, data for knockin clone 1 from Fig. 3 is repeated here for comparison). In addition, co-treatment with the AKT inhibitor GSK690693 resulted in restoration of sensitivity, manifested as synergy between the two compounds. Immunoblot analysis of whole cell lysates shows that treatment of PTEN null cells with lapatinib for 48 h inhibited HER3 phosphorylation (Fig. 4B), as it did in all other tested clones. Treatment of PTEN null cells with lapatinib reduced phospho-AKT levels but not to the levels of parental (see Fig. 2A) or control cells (Fig. 4B). In addition, in the presence of lapatinib, PTEN null cells did not retain levels of phospho-AKT (specifically serine-473) as high as H1047R knockin cells and did not decrease phospho-AKT as dramatically as E545K knockin cells (Fig. 4B). Analysis of time course lysates showed that phospho-AKT levels remained steady over 72 h of exposure to lapatinib. More importantly, levels of phospho-S6 remained high for the duration of lapatinib treatment implying that AKT signaling persists although the upstream stimulus is blunted (Fig. 4C). Taken together with the results from H1047R knockin cells, these data suggest that increased accumulation of PIP_3_—whether by increased kinase activity of PI3-kinase or decreased phosphatase activity by PTEN—may be sufficient for cells to escape the effects of pharmacological inhibition of HER2 signaling.

### Resistance to lapatinib is associated with high levels of membrane-bound PIP_3_

**Fig. 5:**
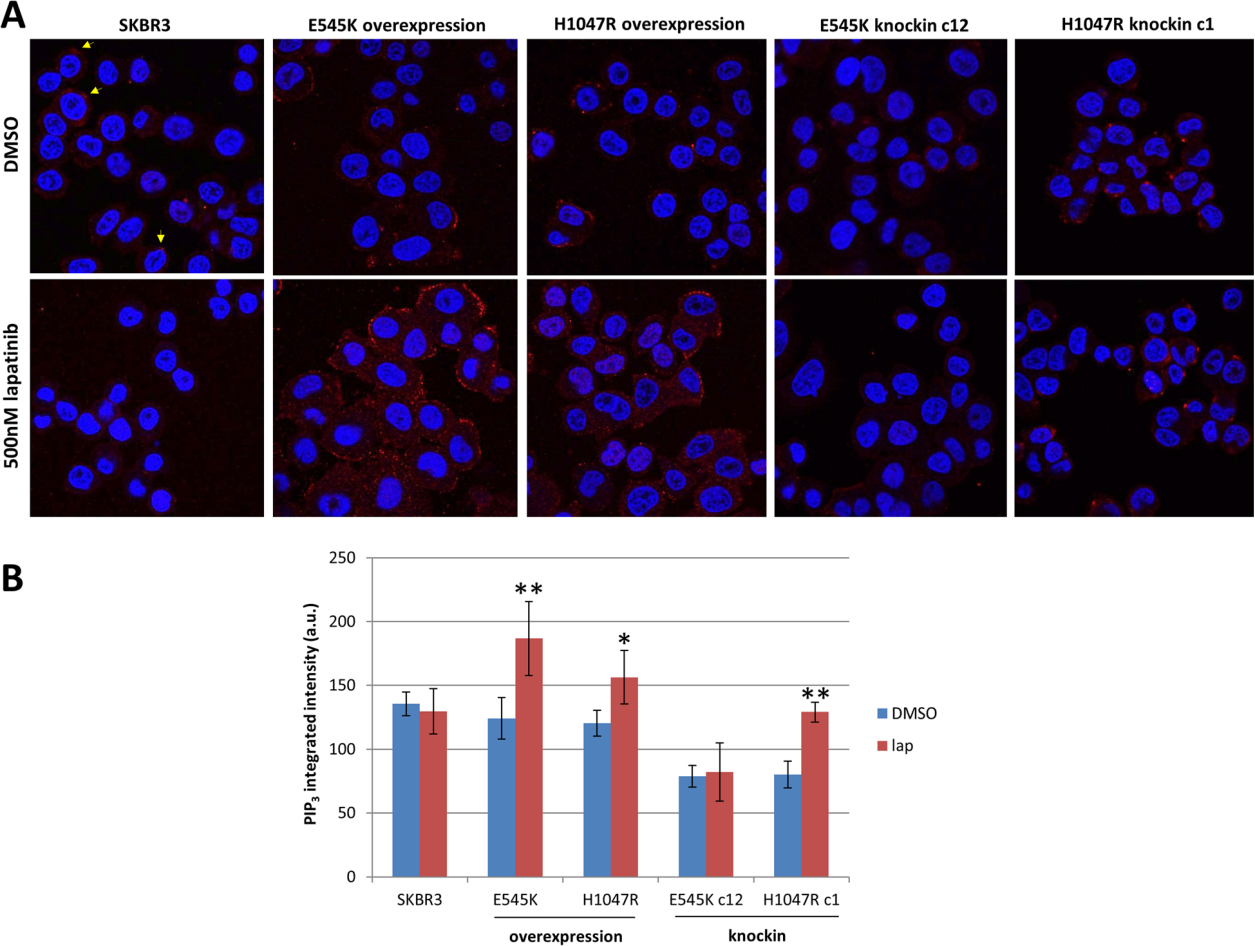
PIP_3_ signaling remains high in H1047R knockin but not E545K knockin cells after lapatinib treatment. **A** Confocal imaging of cells after 36 h of treatment with DMSO or lapatinib (500 nM). PIP3 staining can appear in clusters (yellow arrows, top left image). **B** Quantification of PIP_3_ staining in cells using CellProfiler. Cell boundary was determined by phalloidin stain (not shown) and PIP_3_ stain overlapping with phalloidin was calculated. Sum of pixel intensity of PIP_3_ divided by the area overlapping with phalloidin was calculated for every cell per image. Average values across 15 images are shown in the graph. Error bars represent standard deviation. * and ** indicates PIP_3_ intensity values for lapatinib-treated cells are significantly higher than untreated matched samples (* indicates *p* < 0.005 and ** *p* < 0.001)

We examined levels of PIP_3_ in all our cells because activation of AKT necessarily requires recruitment to the plasma membrane mediated by PIP_3_ [11]. We hypothesized that levels of PIP_3_ have to remain high or even become elevated in cells that show resistance to lapatinib. We used confocal microscopy to quantify membrane-associated PIP_3_ in our knockin and overexpression cells (Fig. 5A). Wildtype SK-BR-3 maintained PIP_3_ levels after treatment with lapatinib (Fig. 5B). In contrast, PIP_3_ levels were significantly elevated after lapatinib treatment in all clones that showed resistance to lapatinib—that is, in clones overexpressing *PIK3CA* mutations and H1047R knockins but not in E545K knockin mutants, which again maintained PIP_3_ at the same level as untreated cells (Fig. 5A, B). Quantification of these results shows a significant increase in PIP3 levels only in H1047R knockin cells and mutant overexpression cells after lapatinib treatment (Fig. 5B).

### Impact of PI3K mutation on response to NRG1β

**Fig. 6:**
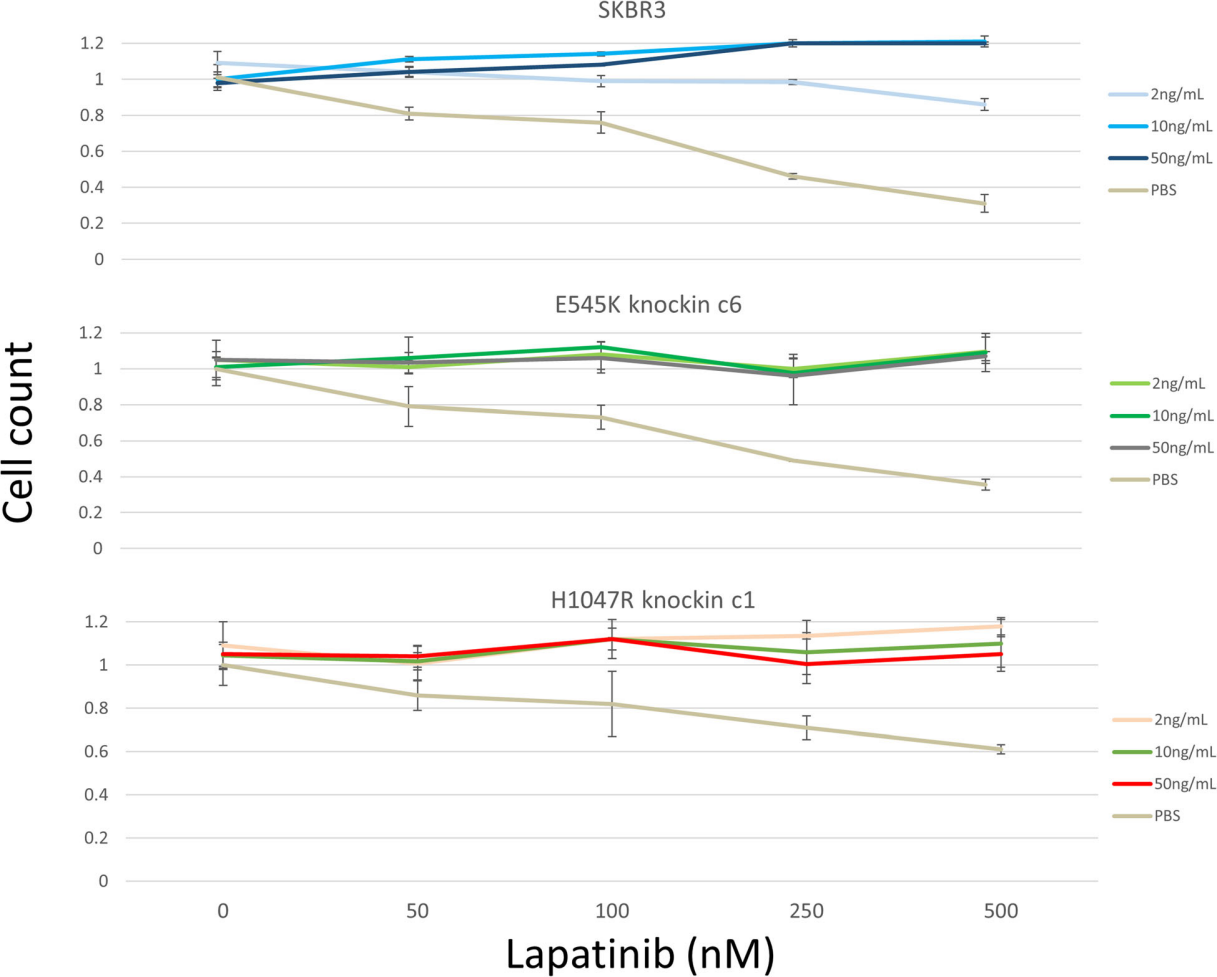
H1047R and E545K knockin cells are hyper-responsive to neuregulin-1 beta 1 (NRG1β) treatment. **A** Parental SK-BR-3 cells treated with NRG1β and lapatinib show rescue from lapatinib at high concentrations of NRG1β, but only partial rescue when treated with low doses of NRG1β (2 ng/mL). **B** The H1047R cells show complete rescue from lapatinib with all doses of NRG1β, even at the highest dose of lapatinib, suggesting that the mutant cells are more responsive to ligand stimulation than their wildtype counterparts. **C** The E545K cells also show complete rescue from lapatinib inhibition by all doses of NRG1β. Value shown are median cell counts relative to untreated controls. Error bars are ± standard deviation

We recently showed that the growth factor neuregulin-1 beta 1 (NRG1β) confers resistance to lapatinib in luminal HER2+ breast cancer cells including SK-BR-3 [36]. We tested the E545K, and H1047R SK-BR-3 knockin cells with NRG1β by treating with fixed doses of NRG1β (0, 2, 10, or 50 ng/ml) both in the presence and absence of four different concentrations of lapatinib. We saw no difference in response to NRG1β as a result of the introduction of the mutations in the absence of lapatinib. However, when lapatinib was present, the cells harboring either mutation were resistant to lapatinib inhibition at a lower dose of NRG1β compared to the wild-type cells (Fig. 6). In the wildtype cells, 2 ng/ml of NRG1β only gave partial resistance to lapatinib, but in both the E545K and H1047R mutant cells, 2 ng/ml of NRG1β conferred complete resistance, even at the very highest dose of lapatinib (500 nM).

### NRG1β interactions with PI3K mutation impact drug response

**Fig. 7:**
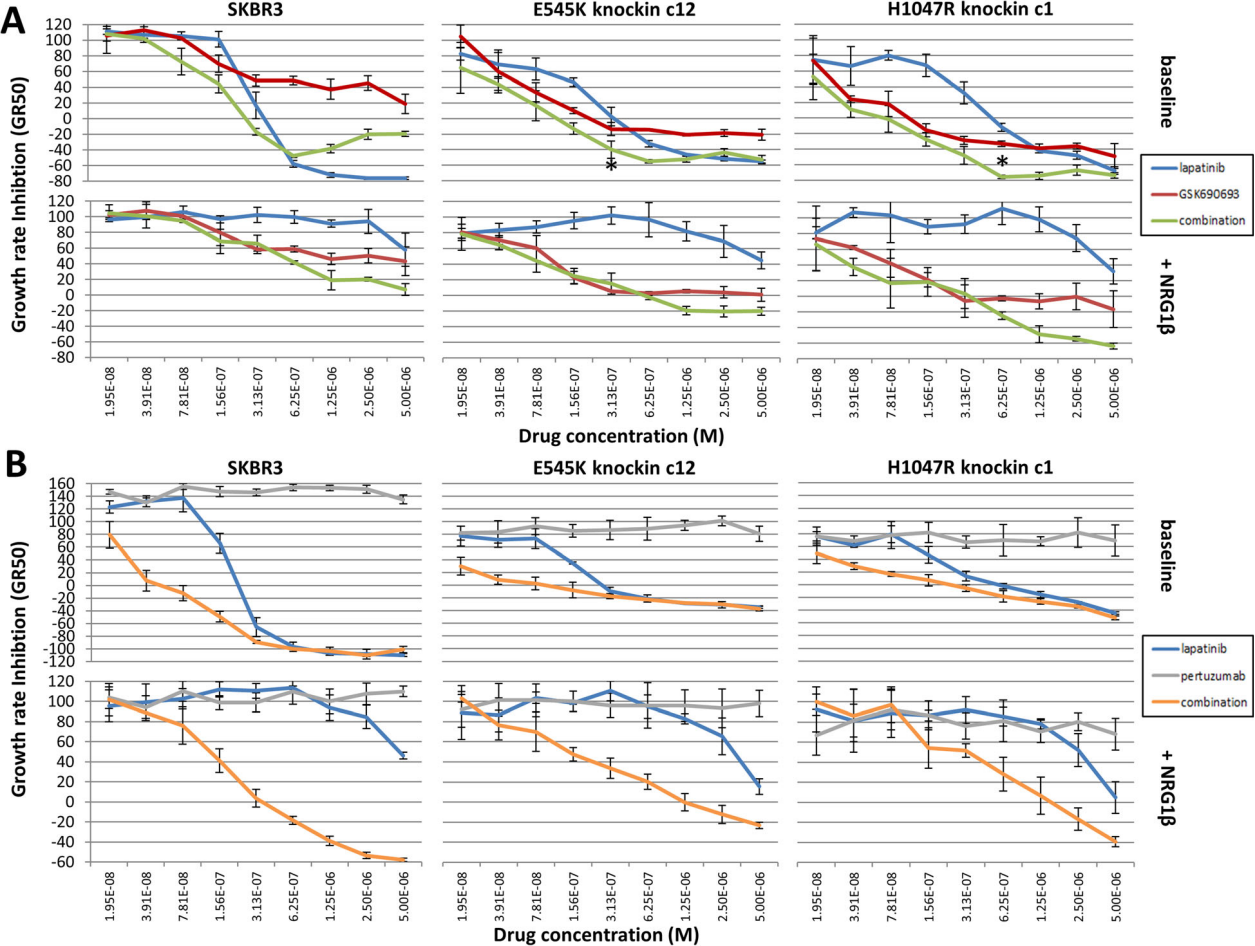
Addition of NRG1β de-sensitizes cells to lapatinib, co-treatment with AKT inhibitor or pertuzumab overcomes this effect. **A** Parental SKBR3 cells or PIK3CA mutant clones treated with lapatinib, AKT inhibitor GSK690693, or combination of the two drugs at a fixed molar ratio in the absence or presence of NRG1β for 72 h. Only H1047R knockin mutant cells show decreased sensitivity to lapatinib at baseline, consistent with results shown in Fig. 1. Treatment with NRG1β makes cells more resistant to lapatinib, and response is partially restored by co-treatment with GSK690693. The efficacy of the combination is greatest in the H1047R mutant cells as measured by the GR_max_ response. Significant synergistic interactions are marked with an *. **B** Parental SKBR3 cells or PIK3CA mutant clones treated with lapatinib, pertuzumab, or combination of the two drugs at a fixed molar ratio in the absence or presence of NRG1β for 72 h. Combination of drugs resulted in synergistic growth inhibition at low doses in all three cell lines, but offered no benefit over lapatinib alone at higher doses. Addition of NRG1β-induced resistance to lapatinib in all three cell lines was abrogated with the addition of pertuzumab. Note that synergy could not be accurately calculated by the combination index method for these curves since it requires fitting dose-response curves and pertuzumab did not have any effect on the growth of cells as a monotherapy

We and others have reported that NRG1β-mediated resistance to lapatinib can be countered using drug combinations, including lapatinib plus pertuzumab [36, 37]. We assessed the impact of the two PI3K mutations on the ability of either pertuzumab or the AKT inhibitor GSK690693 to overcome resistance when used in combination with lapatinib. We first tested lapatinib in combination with GSK690693. As expected, the addition of GSK690693 restored sensitivity to lapatinib in the H1047R mutant cells, resulting in a modest synergistic interaction between the two drugs, which was absent in the wildtype, although some synergy was also seen in the E545K mutant line (Fig. 7A). However, the depth of the response was greatest in the H1047R mutant cell line indicating that these cells are more dependent on PI3K-AKT signaling. When NRG1β was present, the drug combination appeared synergistic in the wildtype and both mutant lines, with the strongest impact in the H1047R mutant line (Fig. 7A), although none reached significance with our stringent measure of synergy. We next tested pertuzumab in combination with lapatinib in the parental, E545K, and H1047R SK-BR-3 cells in the absence of NRG1β. As expected, the mutant cells were more resistant to lapatinib, and pertuzumab on its own had little effect. There appeared to be synergy at the lower end of the concentration curves, but this effect disappeared at higher doses such that lapatinib alone was virtually indistinguishable from lapatinib plus pertuzumab (Fig. 7B). Of note, synergy was impossible to calculate accurately with these treatments, since pertuzumab did not show the dose-response effect that is required for combination index calculations. We next tested the combination when 50 ng/ml NRG1β was present and found again that the combination was able to reverse NRG1β-mediated resistance. The synergy appeared strongest in the wildtype cell line and less significant in the H1047R cell line (Fig. 7B). This suggests that mutation status may also be important in determining the best drug combinations to use to counter NRG1β-mediated resistance, as the AKT inhibitor in combination with lapatinib was more effective in the H1047R cell line while pertuzumab in combination with lapatinib was more effective in the wildtype cells.

### *ERBB2* expression levels in clinical samples are unique between tumors with helical domain mutations and kinase domain mutations

**Fig. 8:**
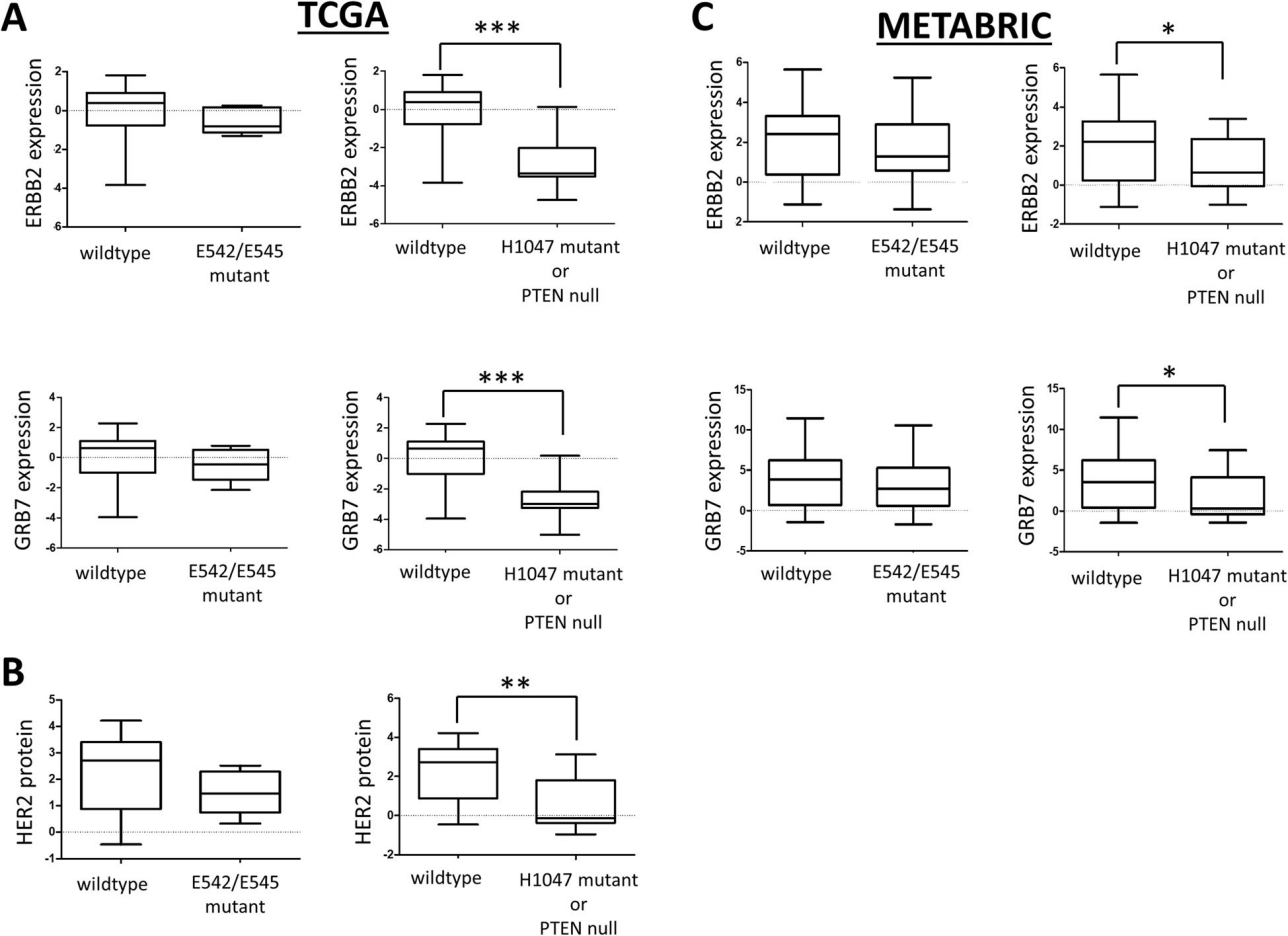
Kinase domain mutant or PTEN-loss samples have significantly lower expression of *ERBB2* and *GRB7*, by Mann-Whitney analysis. **A** Boxplots comparing expression levels of ERBB2 mRNA (top) or GRB7 mRNA (bottom) between wildtype *PIK3CA* samples and either mutant group in RNAseq data from TCGA. ***P value < 0.0001. **B** Boxplots of RPPA data for amount of HER2 protein in wildtype *PIK3CA* samples and either mutant group. *P value < 0.001. **C** Boxplots comparing expression levels of ERBB2 (top) or GRB7 (bottom) between wildtype *PIK3CA* samples and either mutant group in RNAseq data from METABRIC. *P value < 0.01

We explored the clinical significance of our in vitro results by interrogating publicly available datasets. In particular, we were interested to see if levels of expression of *ERBB2* might change in kinase domain mutants, since cells harboring these mutations appear to be less reliant on HER2 to maintain active signaling, and thus there might be less selective pressure to maintain HER2 amplification to high levels. First, we examined the HER2-positive cases from TCGA. Samples were separated into three groups: samples with wildtype *PIK3CA*, samples with mutations in *PIK3CA* at E542 or E545 (helical), and samples with mutations in *PIK3CA* at H1047, and samples with loss of function mutations of PTEN (kinase+PTENloss)—because our results show that PTEN loss phenocopies kinase domain mutations of *PIK3CA*. In the latter two groups, samples with secondary mutations in *PIK3CA* were removed because our in vitro system models each point mutation uniquely. SAM analysis was used to determine genome-wide changes in gene expression between the wildtype and each mutant group. Strikingly, *ERBB2* and *GRB7*—chromosomally co-localized with *ERBB2* and known to be frequently co-amplified with it in HER2-amplified breast cancer [38]—are the two most significantly downregulated genes in the kinase+PTENloss group when compared to wildtype samples (Supplemental Figure 7A). These two genes are not significantly altered in helical domain mutant samples compared to wildtype (Supplemental Figure 7B). Figure 8A shows the boxplot analysis of expression levels of these genes individually. Importantly, protein expression data from TCGA samples corroborate these results (Fig. 8B). That is, samples with H1047 mutation in *PIK3CA* or loss of PTEN have significantly lower protein expression of HER2 compared to wildtype samples, and this is not seen in E542/E545 mutant *PIK3CA* samples. We also note that protein levels of pAKT, pPRAS40, and pS6 are not significantly different between wildtype samples and either the helical group or the kinase+PTENloss group (Supplemental Figure 7C). This underscores the fact that at baseline there is negligible difference in growth/survival pathway signaling in H1047 mutant + PTENloss tumors compared to E545K or wild-type tumors. It is only when the system is perturbed (e.g., blockage of HER2 signaling) that a measurable growth phenotype emerges for the H1047 mutant or PTEN loss samples seen in our in vitro data.

We extended our analysis to the METABRIC dataset [39]. The same three groupings were applied in this dataset and the same boxplot analysis performed on *ERBB2* and *GRB7* expression levels. As seen in TCGA samples, only the kinase+PTENloss group showed statistically significant lower expression of both *ERBB2* and *GRB7* compared to wildtype samples (Fig. 8C). We sought to extend this to further datasets but unfortunately only a limited number of published reports have both RNAseq data and mutational analysis; additionally, those datasets that have both RNAseq and DNA mutation calls are underpowered in terms of *PIK3CA* mutant HER2-enriched samples to perform the same analysis done here [40, 41]. Altogether, our analysis of clinical data show that samples with hotspot mutations in the kinase domain of *PIK3CA* or functional loss of PTEN have lower expression of HER2, both at the mRNA level and protein level.

## Discussion

We generated isogenic knockin mutants of each *PIK3CA* mutation while maintaining the expression of *PIK3CA* under endogenous promoter control to study the impact of mutation on therapeutic response. While our analyses of these cell lines confirm that *PIK3CA* mutations can confer resistance to HER2-targeted kinase inhibitors such as lapatinib and neratinib, we find significant differences in drug response phenotype between the two mutation classes. Specifically, mutation in the helical domain does not confer resistance to lapatinib while mutation in the kinase domain does when *PIK3CA* is expressed at physiological levels. Our time course analysis of lapatinib-treated cells shows striking differences in AKT phosphorylation levels between the different mutants. Lapatinib does not durably suppress PI3K-AKT signaling in H1047R knockin mutant cells for much more than 12 h, but does suppress pAKT in E545K knockin mutants for at least 72 h. As a result, AKT signaling is maintained in cells carrying H1047R mutations, allowing the cell to escape apoptosis via canonical signaling through mTORC and ribosomal protein S6. Thus, the kinase domain mutation confers resistance by causing sustained AKT signaling and associated high levels of PIP_3_ in the presence of HER2 inhibitors. These kinase domain mutant cells respond synergistically to treatment with lapatinib and the pan-AKT inhibitor GSK690693. Cells carrying E545K mutations are not able to maintain PIP3 signaling in the presence of lapatinib and remain sensitive to lapatinib, and treatment with GSK690693 does not increase efficacy. Importantly, these results suggest that H1047R mutants are capable of maintaining signaling independent of signals from the receptor, whereas E545K mutants are not.

These results are quantitatively different than those obtained by overexpressing the *PIK3CA* mutations, which showed that both mutations increased resistance to lapatinib. The increased resistance to lapatinib observed in both of the overexpression clones suggests that introducing oncogenic mutations via overexpression may mask the true biology seen in cells in which a single mutant allele is driven from the endogenous promoter—the genotype that is most commonly seen in mutations of oncogenes. However, as we found that if we overexpressed the E545K mutant using retroviral vectors, we could achieve the same levels of resistance as observed in the H1047R mutants; it suggests that if the E545K mutation is also overexpressed in a tumor, it would likely cause resistance to therapy. Thus, our findings support the notion that if cells express the E545K mutants at physiological levels, then they are unlikely to be resistant to therapy, but overexpression of the same mutant will result in resistance. In contrast, H1047R mutants will result in resistance regardless of the level of expression.

Cells engineered to carry the H1047R kinase domain mutation upregulated PIP_3_ during treatment with high doses of HER2-targeted therapy such as lapatinib. High levels of PIP_3_ maintained high levels of phospho-AKT and hence activation of ribosomal protein S6. Active S6 combines with other proteins to form the 40S ribosomal subunit that then carries out translation initiation [42]. Cells carrying the E545K helical domain mutation did not upregulate high PIP_3_ levels during lapatinib treatment and remained sensitive to the drug. Although the mechanism by which this occurs remains unknown, we speculate that the kinase domain mutation decouples the PI3K pathway from the receptor, allowing it to function independently if growth signals from HER2 are removed. Thus, when HER2 is inhibited, the H1047R mutant of PIK3CA is able to compensate through increased activity, resulting in increased PIP_3_ levels. In contrast, the E545K mutant still relies on signal from HER2 for activity, and thus is unable to increase PIP_3_ to maintain signaling in the same manner. We showed that PI3-kinase is the signaling axis utilized in these cells since inhibition of the Ras-Raf-MEK-ERK axis had no effect on cell growth. Changes in other downstream targets of phospho-AKT signaling such as phosphorylation levels of 4E-BP-1 were not observed. This is not surprising because phosphorylation of 4E- BP-1 at Thr37/46 is insensitive to rapamycin [43] and so it is possible that it is also insensitive to other perturbations of mTORC1 signaling investigated in this study.

We and others have shown previously that NRG1β interacts with the HER2/3 heterodimer to induce resistance to lapatinib and neratinib [36]. We show now that the E545K and H1047R knockin cells became hypersensitized to NRG1β-mediated resistance to lapatinib, as lower levels of NRG1β were capable of conferring resistance to lapatinib than in wildtype cells. This potentially has important implications for patients, since the kinase domain mutation could impact lapatinib response on multiple levels—first by making cells more resistant at baseline, and second by making them more susceptible to NRG1β-mediated changes in HER2/3 heterodimer conformation that reduces the binding efficacy of lapatinib and neratinib. We previously reported that serum levels of NRG1β equivalent to the doses used in these studies can be found in patients [36], suggesting that these effects are operational in the clinic. Indeed, high levels of NRG1β in all HER2+ breast cancer patients were reported to be correlated with higher rates of recurrence [44], which may be exacerbated by the presence of PIK3CA mutations.

The differences in response phenotype between the kinase and helical domain mutants have not been observed before because the vast majority of previous studies have used traditional transgene overexpression to study the function of mutations of *PIK3CA*. Our results support previous reports showing the value of knockin techniques to uncover subtle phenotypes that might otherwise be obfuscated by traditional transgene overexpression [23, 30]. Slight differences in p110α kinase activity have been previously reported for kinase and helical domain mutations [20]. However, our report is the first to show differential response to drug because of the proposed lower catalytic activity of the E545K mutation compared to H1047R mutation when both are expressed at physiological levels. Additionally, our results provide a functional definition for the term synergy in this particular case of drug combinations; specifically, in a lapatinib-resistant cell line, co-treatment with an AKT inhibitor and lapatinib restores the effect of drug to what is seen with lapatinib alone seen in wildtype cells.

Additionally, we show here that unique gene expression patterns exist depending on the *PIK3CA* mutation. We speculated that because the H1047R mutants appear to be less reliant on signaling from the receptor, there might be less selective pressure to maintain high levels of HER2. Indeed, we found that the kinase domain and PTEN mutants had lower levels of expression of both HER2 and GRB7 in two large independent public data sets, whereas the helical domain mutants did not show this difference. Kinase domain mutant cells may not respond well to HER2-targeted therapy as there is significant downregulation of the gene *ERBB2* in these clinical samples and concomitant lower protein expression of HER2 not seen in helical mutant samples in addition to the PI3K mutation; this hypothesis is bolstered by published evidence that high levels of HER2 expression are predictive of response to lapatinib and trastuzumab [45]. While it is impossible to know the sequence of genomic alteration in these tumors—that is, whether HER2 became amplified first or *PIK3CA* acquired its mutation first—these findings may suggest a mechanism by which patients acquire resistance even prior to therapy. Specifically, the genomic locus of HER2 is amplified which is detected clinically at the DNA level and informs the clinician to start HER2-targeted therapy, but *PIK3CA* also acquires a mutation in the kinase domain; this kinase mutation renders the protein constitutively active which circumvents the need for continuous signaling from HER2, causing downregulation of HER2 expression at the mRNA level and the protein level, thereby making the tumor refractory to HER2-targeted therapy. While this mechanism is speculative, the data we present here clearly show that the two hotspot mutations of *PIK3CA* respond differently to HER2-targeted therapy in vitro and that clinical samples of HER2-amplified tumors with *PIK3CA* mutation have unique gene expression patterns which support our results.

Ultimately, the phenotypic disparity between helical and kinase domain mutations of *PIK3CA* may have important clinical implications. It is important to note that publicly available data [46, 47] demonstrates that approximately 21% of HER2-amplified cases of breast cancer also harbor a mutation at one of the hotspots of *PIK3CA.* Our results suggest that HER2+ tumors with helical domain mutations of *PIK3CA* will respond to tyrosine kinase inhibitors such as lapatinib and neratinib whereas HER2+ tumors with H1047R mutations will not, although both a hypersensitive to resistance mediated by NRG1β. Our data and previous studies also suggest that adding pertuzumab to counter NRG1β-mediated resistance to lapatinib and neratinib will be important in HER2+ tumors carrying *PIK3CA* mutations of either type. Altogether, we show that important phenotypic disparities exist between the two hotspot mutations of *PIK3CA*. These data raise the intriguing possibility that patients may one day benefit from differential treatments based on PIK3CA mutational status. Our study sets the stage for the necessary pre-clinical animal studies and follow-up clinical investigations to validate these findings.

## Supplementary Information

**Additional file 1: Supplemental Figure 1.** Analysis of clones overexpressing transgenic PIK3CA reveals supraphysiological levels of mutant allele. **Supplemental Figure 2.** Results of continuous live-cell imaging. **Supplemental Figure 3.** Response to the pan-AKT inhibitor GSK690693. **Supplemental Figure 4.** Synergistic response with combinatorial drug treatment in H1047R knockin clones. **Supplemental Figure 5.** MEK inhibition does not synergize with inhibition of HER2 signaling in HER2-amplified cells. **Supplemental Figure 6.** Validation of PTEN knockout in two SKBR3 clones. **Supplemental Figure 7.** Significance analysis of microarrays (SAM) results.

**Additional file 2: Supplemental Table 1.** List of primers used in this study. **Supplemental Table 2.** List of antibodies used in this study.

## Data Availability

The datasets used and/or analyzed during the current study are available from the corresponding author on reasonable request.
