# Supplementary material for: Sensitivity to targeted therapy differs between HER2-amplified breast cancer cells harboring kinase and helical domain mutations in PIK3CA
Source: Breast Cancer Res. 2021 Aug 3;23:81. doi: 10.1186/s13058-021-01457-0 (PMC8336338; doi:10.1186/s13058-021-01457-0)
Supplement: Supplementary file 1 — Additional file 1: Supplemental Figure 1. Analysis of clones overexpressing transgenic PIK3CA reveals supraphysiological levels of mutant allele. Supplemental Figure 2. Results of continuous live-cell imaging. Supplemental Figure 3. Response to the pan-AKT inhibitor GSK690693. Supplemental Figure 4. Synergistic response with combinatorial drug treatment in H1047R knockin clones. Supplemental Figure 5. MEK inhibition does not synergize with inhibition of HER2 signaling in HER2-amplified cells. Supplemental Figure 6. Validation of PTEN knockout in two SKBR3 clones. Supplemental Figure 7. Significance analysis of microarrays (SAM) results. [file 13058_2021_1457_MOESM1_ESM.pdf]

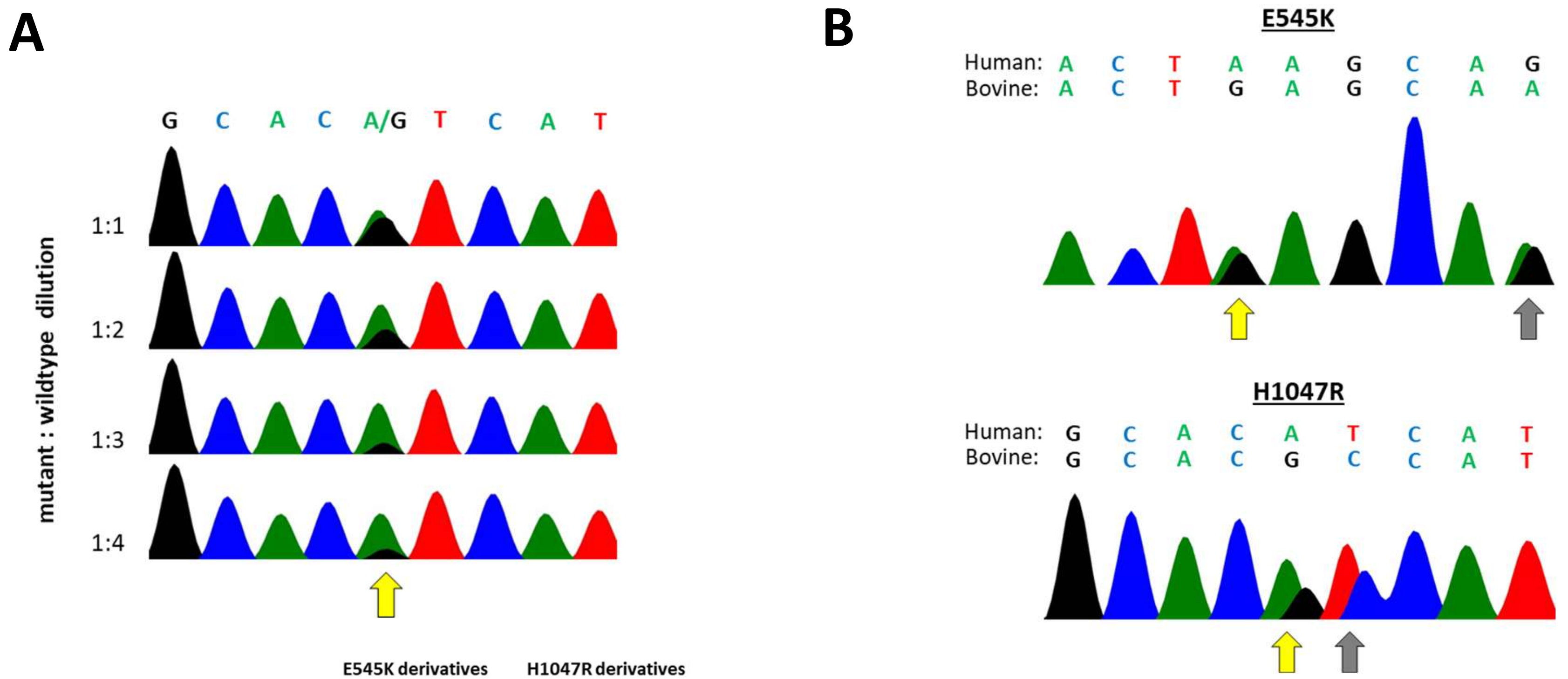

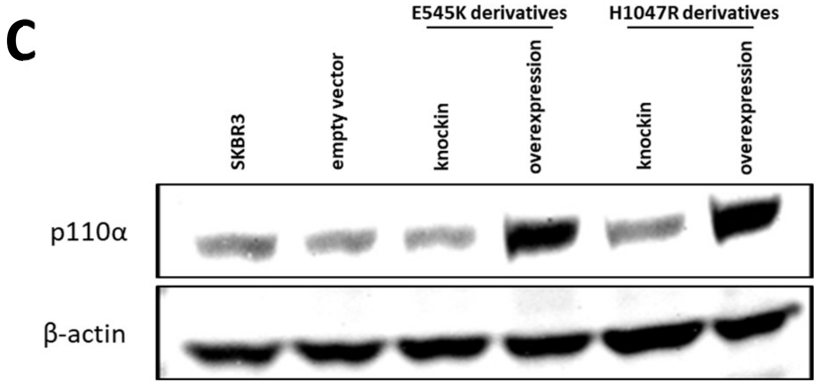

Supplemental Figure 1. Analysis of clones overexpressing transgenic PIK3CA reveals supraphysiological levels of mutant allele.

A. Sequencing of known wildtype or mutant allele PCR fragments mixed at designated ratios shows that Sanger sequencing offers an approximation of allele ratios in a sample based on the height of the corresponding peak in sequencing traces (yellow arrows).

B. Targeted Sanger sequencing of cDNA of SKBR3 overexpression clones shows multiple copies of mutant transgene in cells (yellow arrows). Recombinant bovine PIK3CA coding sequence with either E545K or H1047R mutation was used as the cDNA construct for transgene overexpression of PIK3CA in cells. Single nucleotide polymorphisms between human and bovine PIK3CA coding sequence serve as internal controls for estimation of allele frequency (gray arrows).

C. Western blot analysis of whole cell lysates from SKBR3 cells and PIK3CA mutant clones show increased expression of p110α only in overexpression clones. In silico analysis of bovine and human PIK3CA shows 99% homology between the two proteins demonstrating that the antibody can recognize the recombinant overexpressed version. Beta-actin serves as a protein loading control.

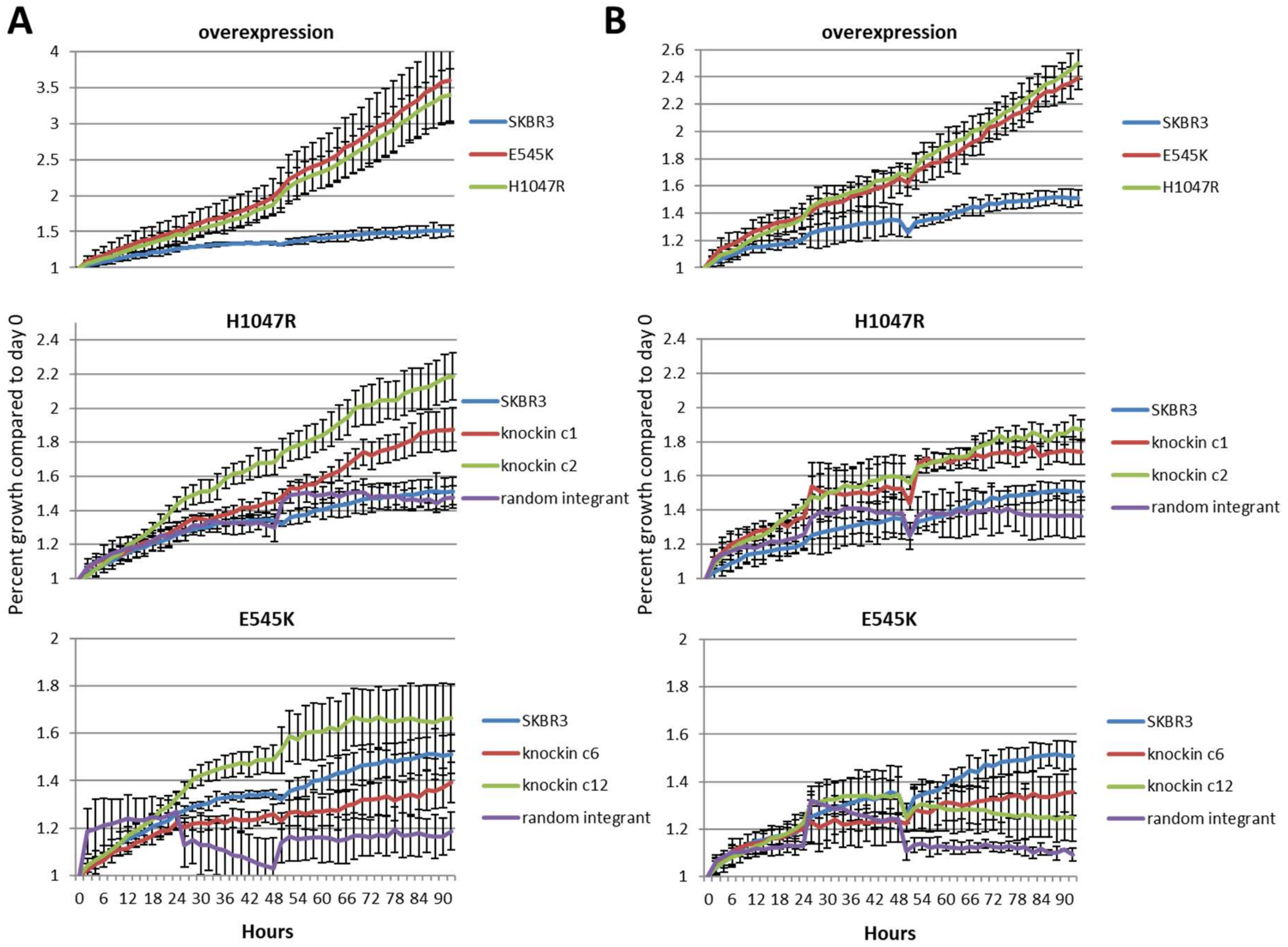

Supplemental Figure 2. Continuous live-cell imaging of cells exposed to 500nM lapatinib (A) or 50nM neratinib (B) for 90 hours. Medium and drug was replenished every 24 hours for the duration of the assay. Error bars represent standard deviation for triplicate values.

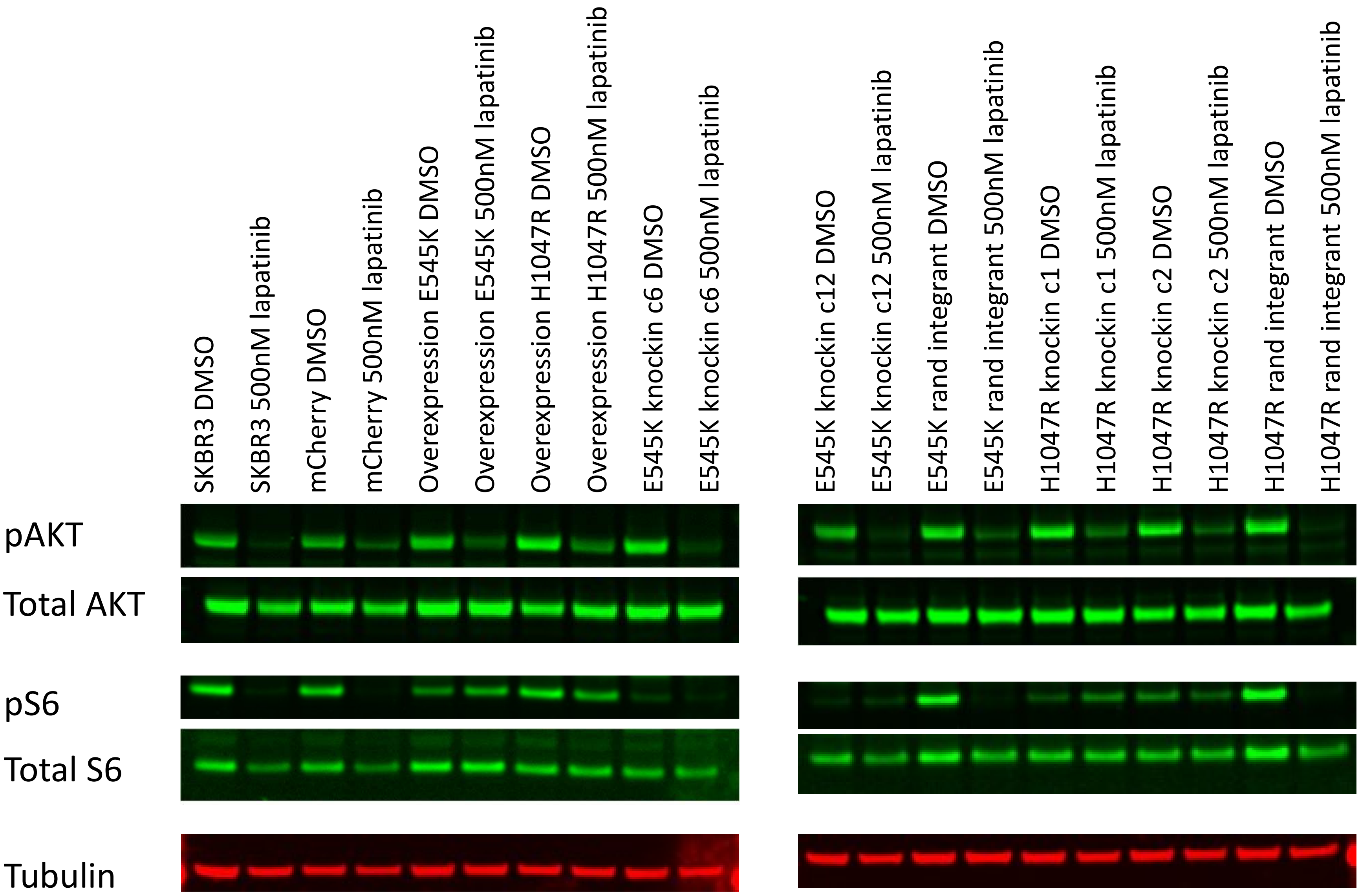

Supplemental Figure 2C. Example Western blots used to generate data for figure 2A showing loading order on gels.

A

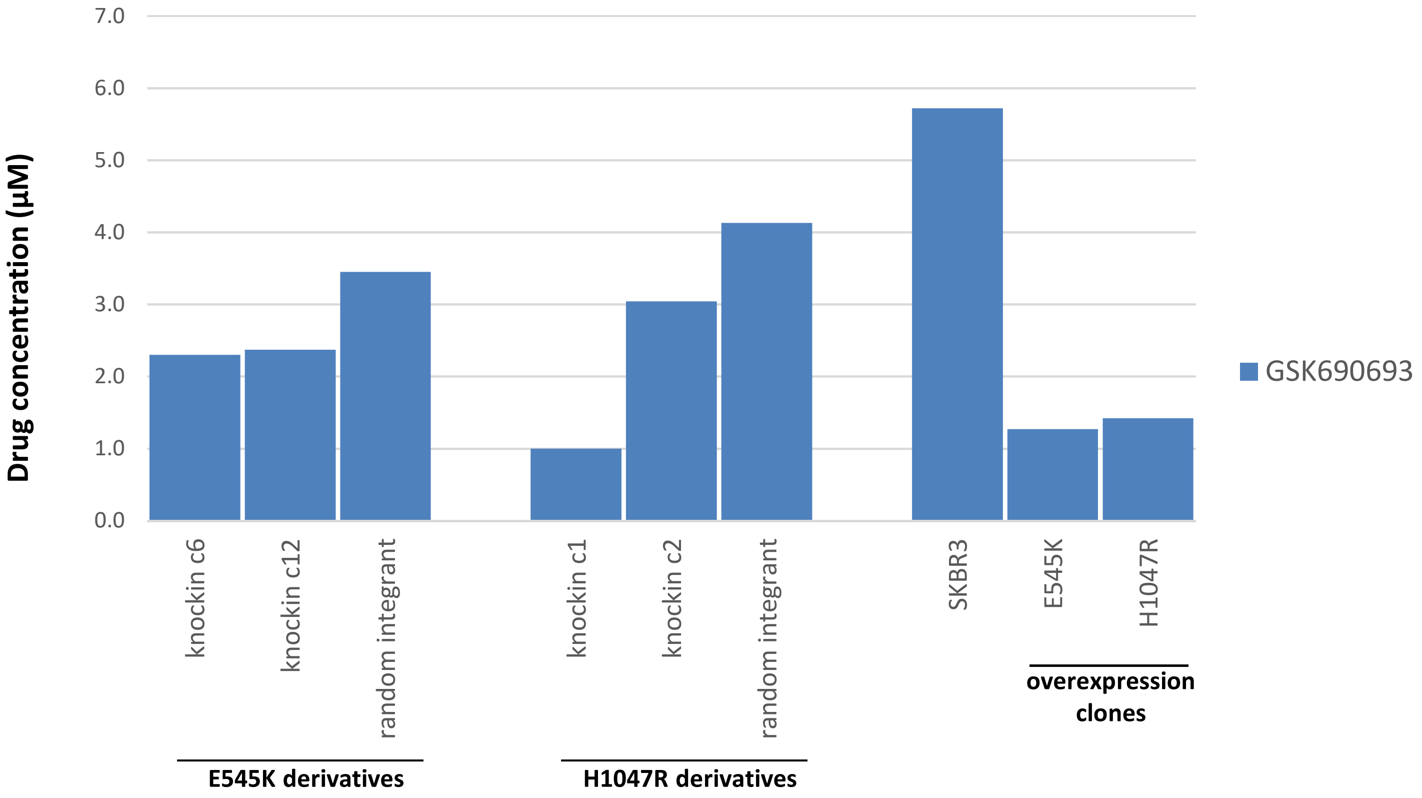

B

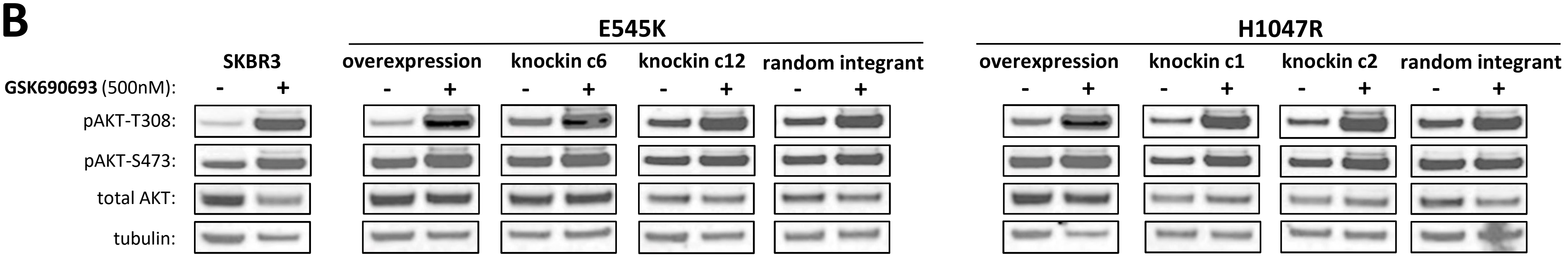

Supplemental Figure 3. Response to the pan-AKT inhibitor GSK690693

A. GR50 calculations for all cell lines show that knockin derivatives and overexpression clones for each PIK3CA mutation maintain sensitivity to the pan-AKT inhibitor GSK690693 in the micromolar range. The data shown derive from one of three biological replicates. B. Whole cell lysates collected after 48 hours of exposure to 500nM GSK690693 or vehicle were separated by gel electrophoresis and analyzed for the indicated proteins. C. All Western blots, including those showing decrease in pRAS40-S346 and pS6-235/6 in all cells following treatment with the AKT inhibitor GSK690693 (following 3 pages).

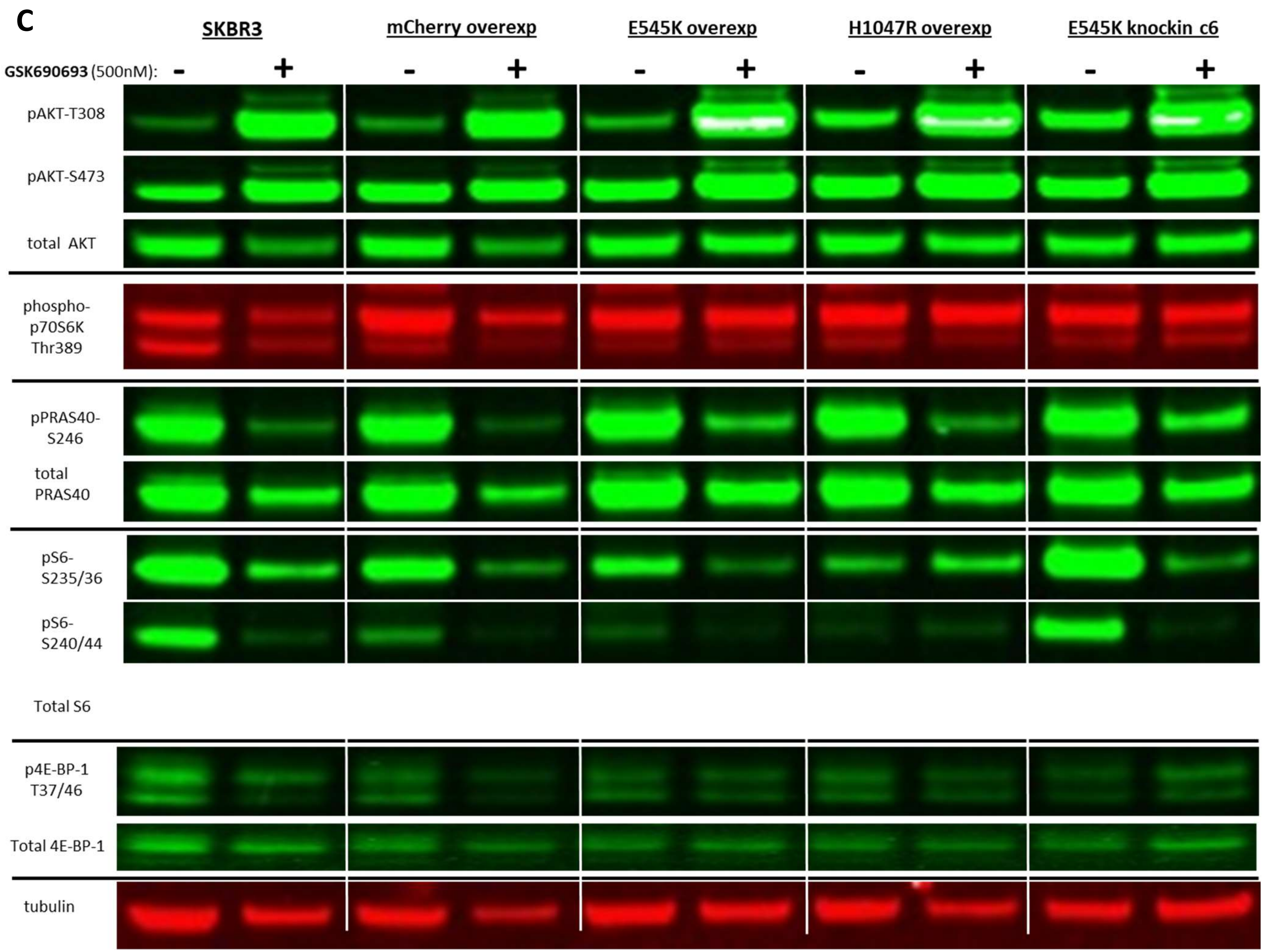
C
SKBR3
mCherry overexp
E545K overexp
H1047R overexp
E545K knockin c6
GSK690693 (500nM): - + - + - + - + - +
pAKT-T308
pAKT-S473
total AKT
phospho-p70S6K Thr389
pPRAS40-S246
total PRAS40
pS6-S235/36
pS6-S240/44
Total S6
p4E-BP-1 T37/46
Total 4E-BP-1
tubulin

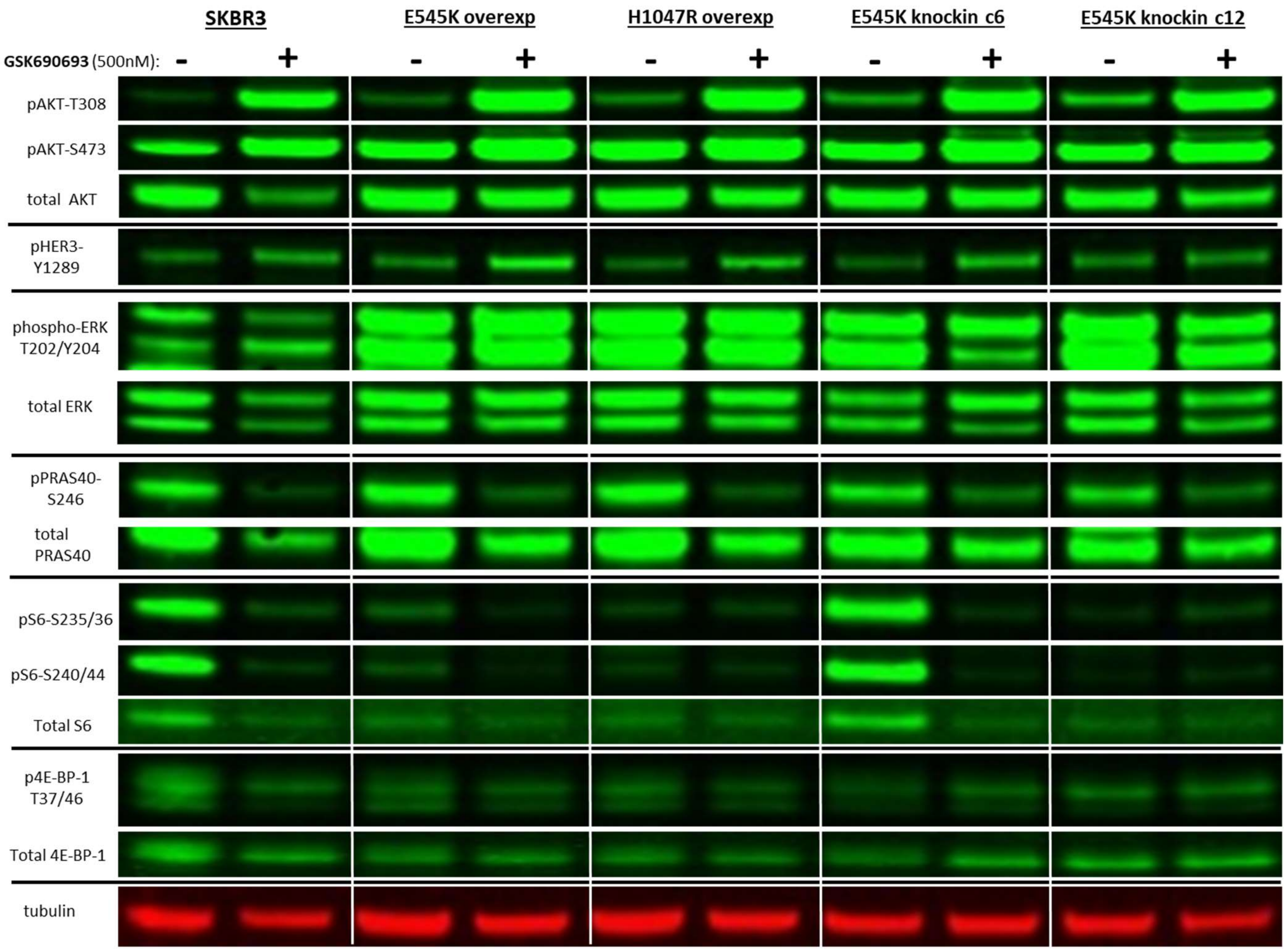
SKBR3
E545K overexp
H1047R overexp
E545K knockin c6
E545K knockin c12
GSK690693 (500nM): - + - + - + - + - +
pAKT-T308
pAKT-S473
total AKT
pHER3-Y1289
phospho-ERK T202/Y204
total ERK
pPRAS40-S246
total PRAS40
pS6-S235/36
pS6-S240/44
Total S6
p4E-BP-1 T37/46
Total 4E-BP-1
tubulin

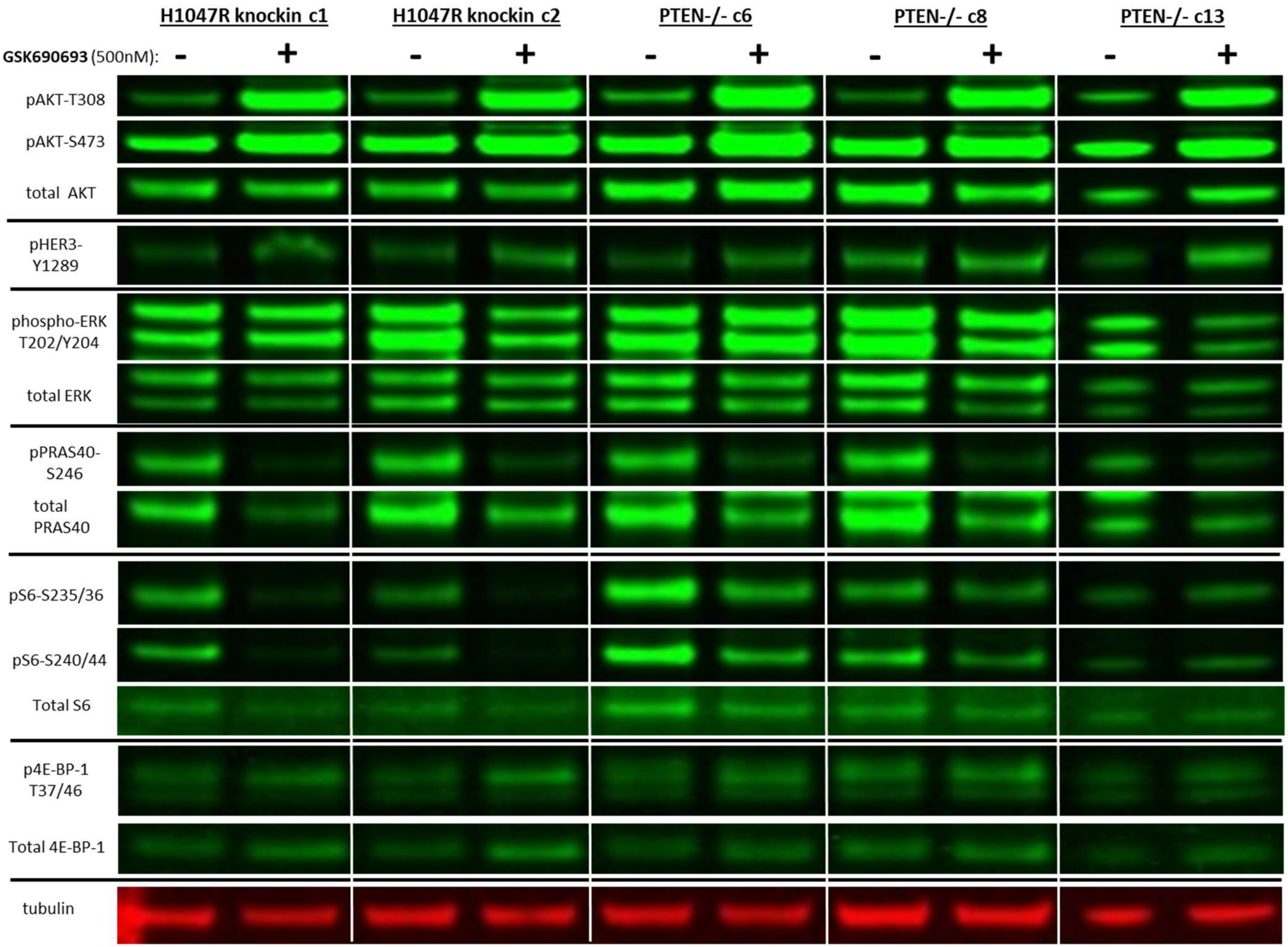
H1047R knockin c1
H1047R knockin c2
PTEN-/- c6
PTEN-/- c8
PTEN-/- c13
GSK690693 (500nM):
-
+
pAKT-T308
pAKT-S473
total AKT
pHER3-Y1289
phospho-ERK T202/Y204
total ERK
pPRAS40-S246
total PRAS40
pS6-S235/36
pS6-S240/44
Total S6
p4E-BP-1 T37/46
Total 4E-BP-1
tubulin

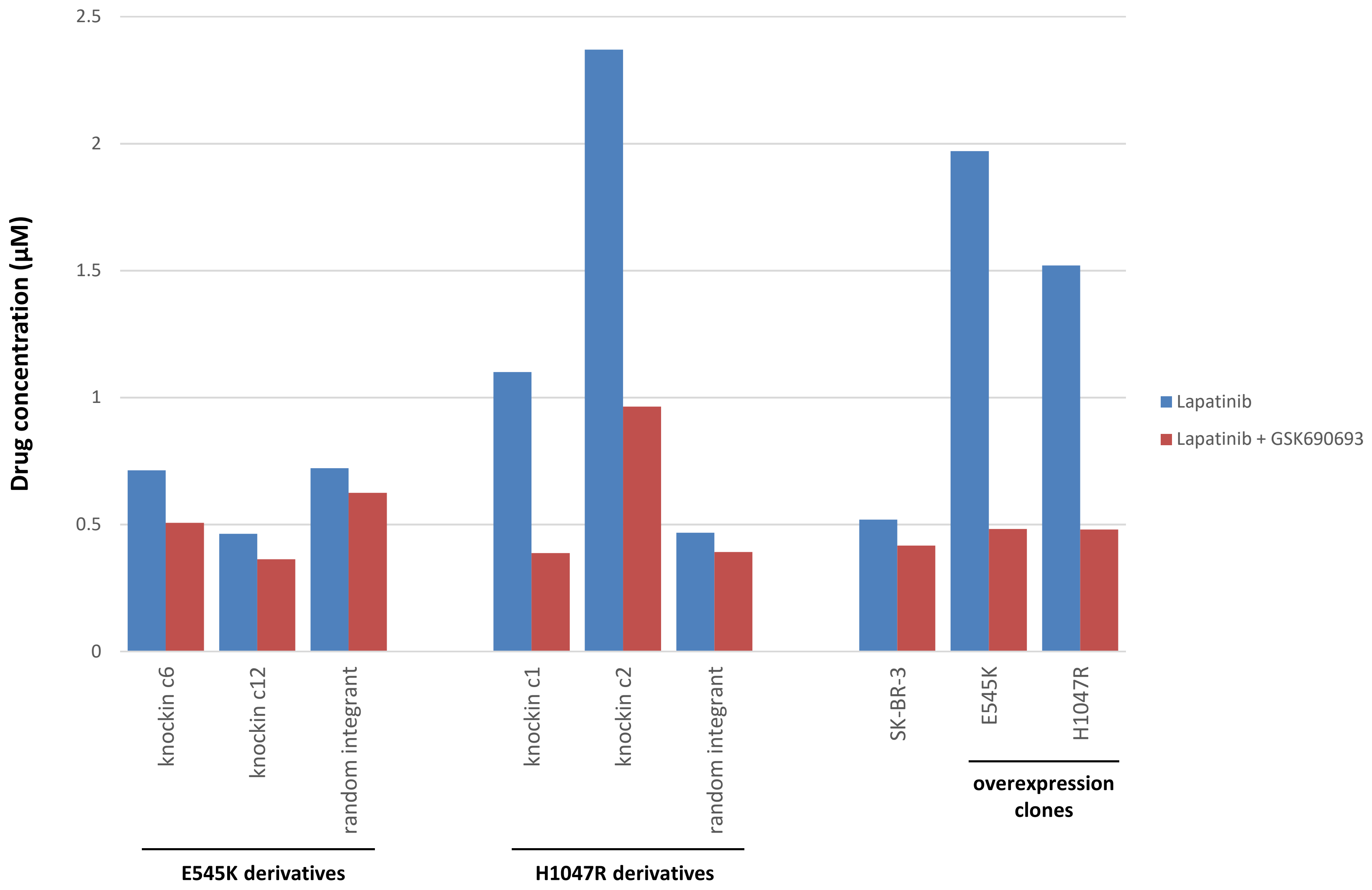

Supplemental Figure 4. Beneficial effects of combinatorial drug treatment in H1047R knockin clones. GR50 calculations for all cell lines show that only H1047R knockin derivatives are insensitive to lapatinib monotherapy but show significant reduction when the pan-AKT inhibitor GSK690693 is added to lapatinib treatment. Both mutant overexpression clones also show this effect highlighting how transgenic overexpression can mask biological phenotypes. The data shown derive from one of three biological replicates.

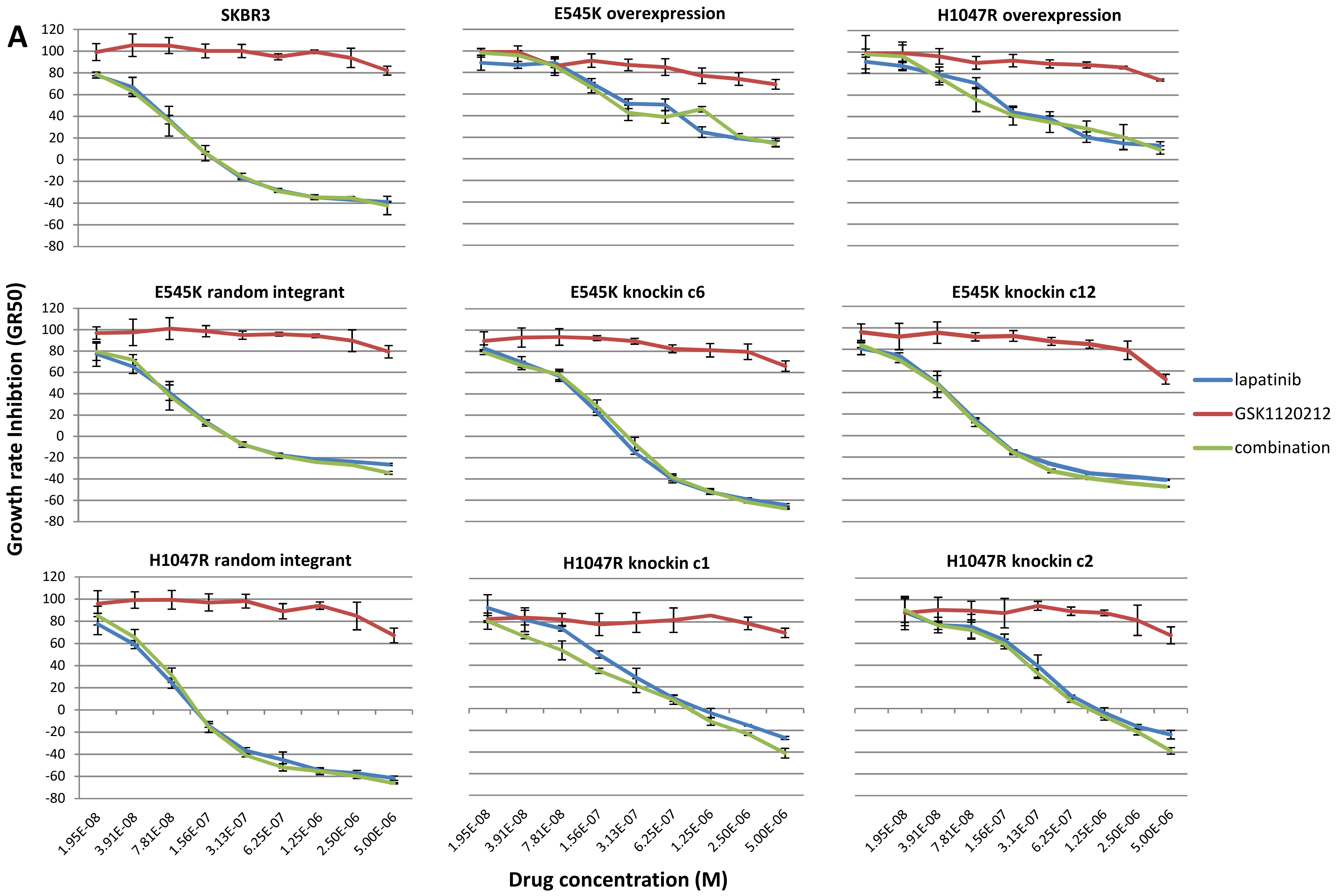

Supplemental Figure 5A. MEK inhibition does not synergize with inhibition of HER2 signaling in HER2-amplified cells. Cells treated for 72 hours with 2-fold dilutions of lapatinib (blue), MEK inhibitor GSK1120212 (red), or equimolar combinations of both (green).

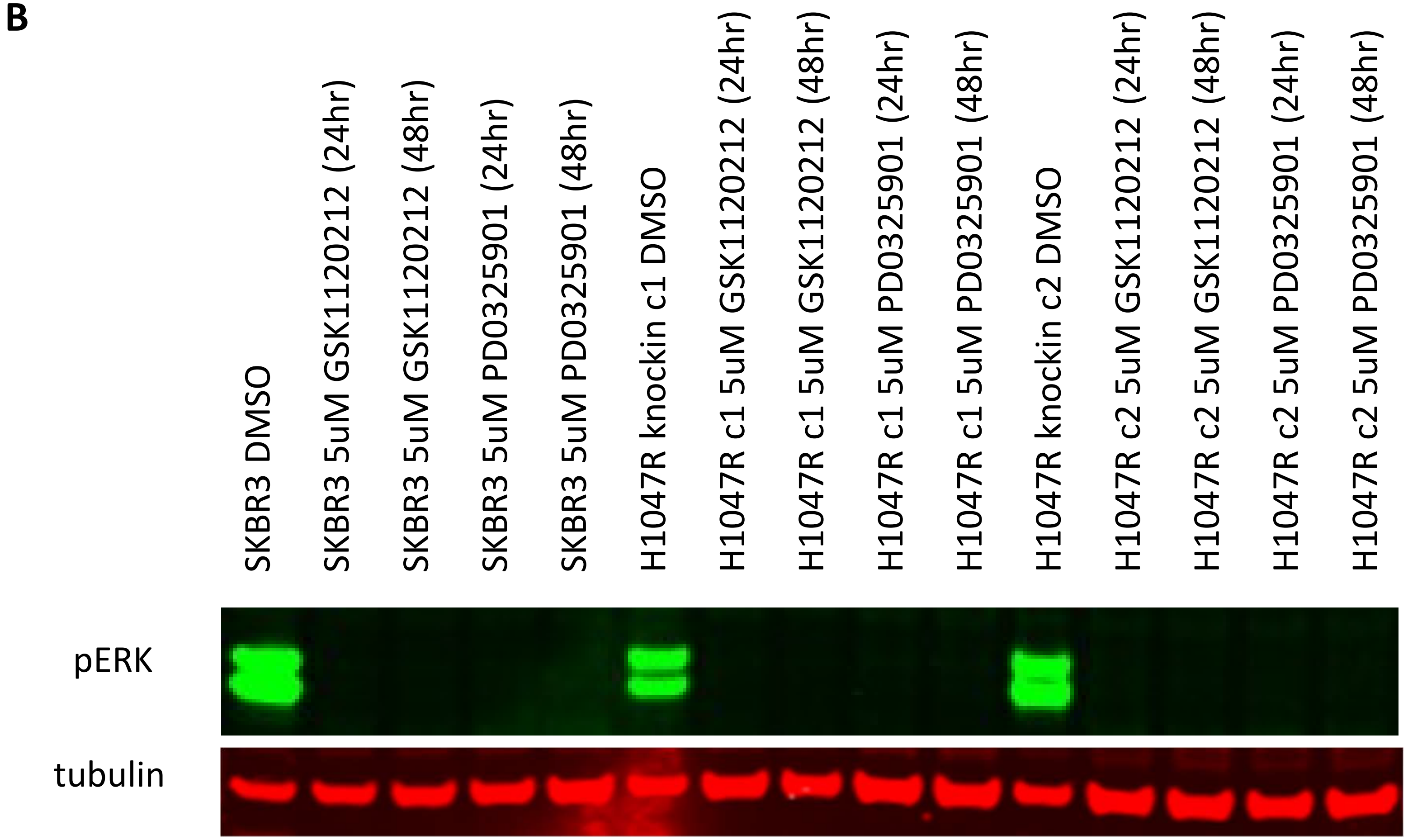

Supplemental Figure 5B. Treatment of wild-type and mutant cells with MEK inhibitors GSK1120212 or PD0325901 results in durable suppression of p-ERK.

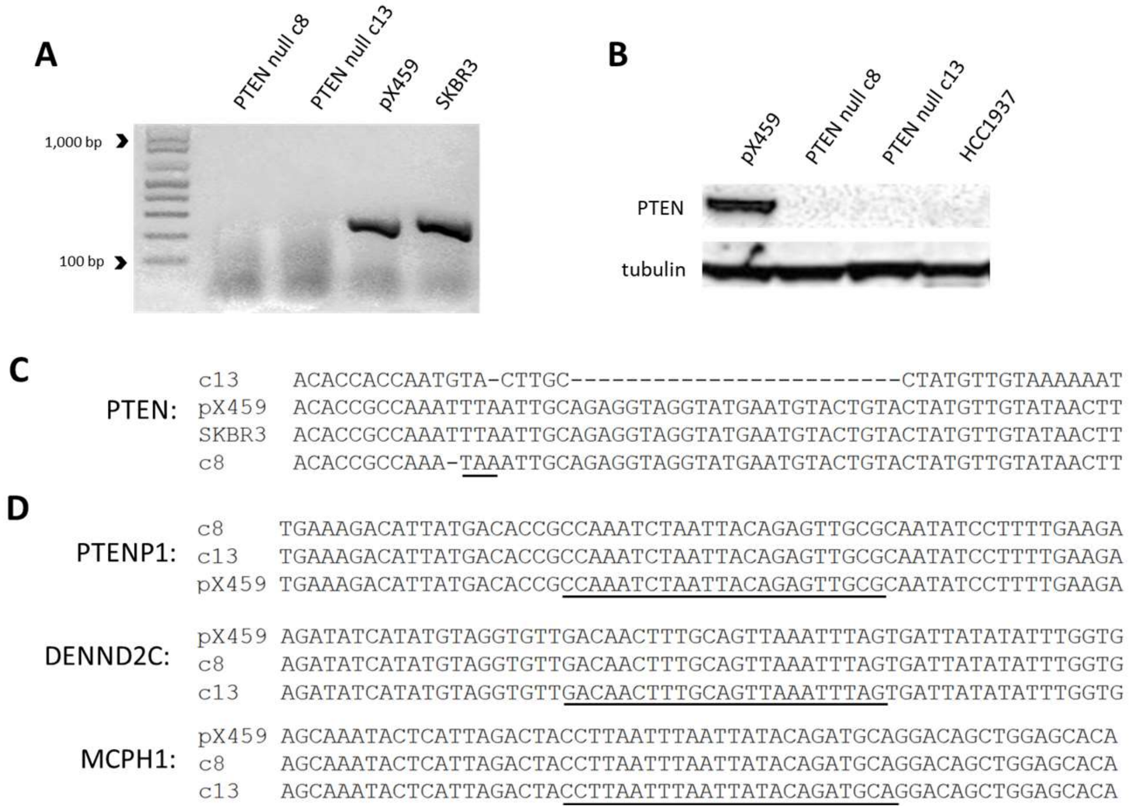

Supplemental Figure 6. Validation of PTEN knockout in two SKBR3 clones.

A. RT-PCR for PTEN using exon-spanning primers. pX459 is the empty vector CRISPR construct used as control.
B. Western blot analysis of whole cell lysates from PTEN-/- SKBR3 cells and controls. Breast cancer cell lines HCC1937 is null for PTEN expression and was included as an additional control.
C. Multiple sequence alignment of the PTEN locus targeted by CRISPR. In clone 8, cutting by Cas9 and repair by endogenous proteins removes a single base pair creating a frameshift that results in a stop codon in the new reading frame immediately following cut site (underlined). In clone 13, Cas9 cutting results in loss of 25 base pairs which creates a stop codon approximately 11 amino acids downstream of the region shown.
D. Multiple sequence alignment of the top three off-target sites for the PTEN CRISPR. The proposed sequence recognized by PTEN CRISPR is underlined in each alignment.

A

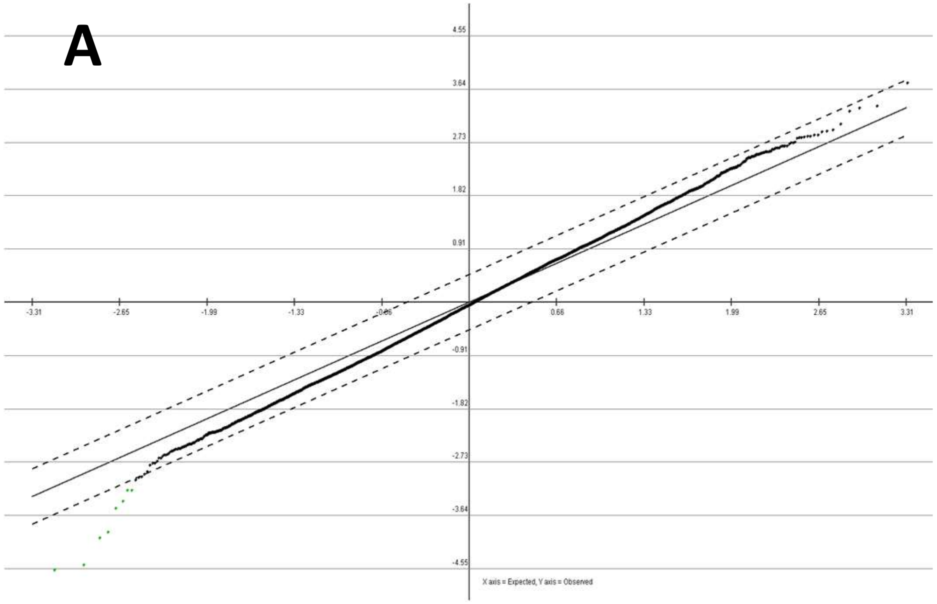

	Expected Score (dExp)	Observed Score (d)	Fold Change (unlogged)
ERBB2|2064	-2.5697343	-4.55092	0.26247
GRB7|2886	-1.1640501	-4.4663267	0.24254546
HFE|3077	0.37239385	-3.9988515	0.37975118
BCAS1|8537	-0.05706396	-3.900895	0.31524855
STARD3|10948	-1.2751213	-3.5009668	0.4467569
PGAP3|93210	-1.312751	-3.3790438	0.35627747
SYT17|51760	0.27216232	-3.1900132	0.5492354
C17orf37|84299	-1.3333074	-3.185001	0.58102834

B

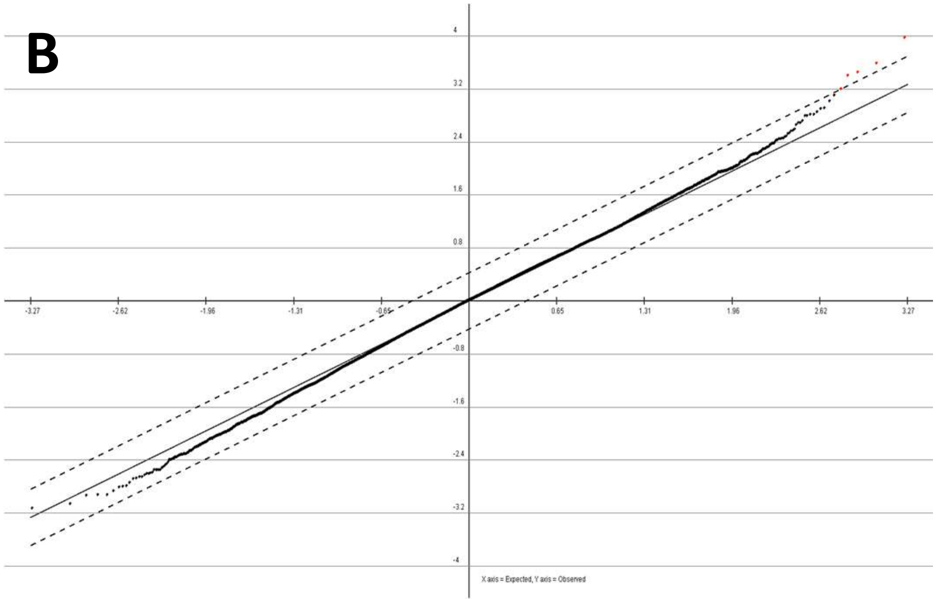

	Expected Score (dExp)	Observed Score (d)	Fold Change (unlogged)
SCNN1G|6340	0.9870989	3.5431921	7.113985
GRIK4|2900	1.4671634	4.0312247	5.805726
NKAIN1|79570	0.5849106	3.4129121	4.8171625
MS4A15|219995	1.2089868	3.2672873	3.9685805
MCHR1|2847	1.125369	3.3179657	2.7732637

Supplemental Figure 7. Significance analysis of microarrays (SAM) results of HER2-enriched TCGA samples show unique differentially expressed genes between H1047-mutant samples and E542/E545-mutant groups.

A. Results of two-class unpaired SAM test of all samples using wildtype *PIK3CA* (group 1) versus samples with either mutation at H1047 in *PIK3CA* or loss of function mutation in *PTEN* (group 2). The significantly altered genes are shown below. False discovery rate = 0.
B. Results of two-class unpaired SAM test of all samples using wildtype *PIK3CA* (group 1) versus samples with mutation at E542 or E545 in *PIK3CA* (group 2). The significantly altered genes are shown below. False discovery rate = 0.

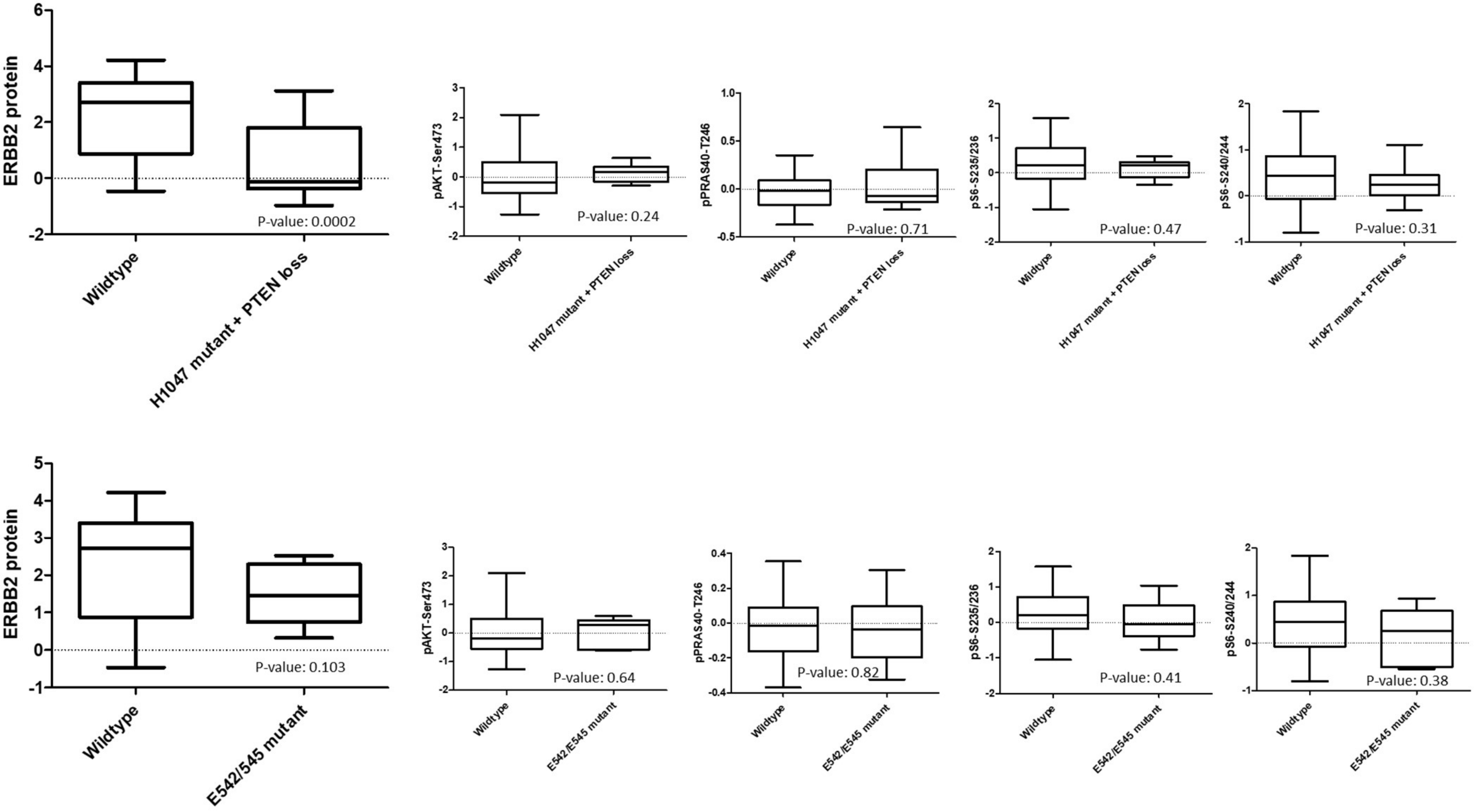

Supplemental Figure 7C. Expression of pAKT, pPRAS40, and pS6-S235/2366 or pS6-S240/244 do not show significant differences in protein expression between either H1047R/PTEN mutants or E545K mutants and wild-type cells.
